# In Vitro Evaluation of New 5-Nitroindazolin-3-one Derivatives as Promising Agents against *Trypanosoma cruzi*

**DOI:** 10.3390/ijms252011107

**Published:** 2024-10-16

**Authors:** Josué Pozo-Martínez, Vicente J. Arán, Matías Zúñiga-Bustos, Sebastián Parra-Magna, Esteban Rocha-Valderrama, Ana Liempi, Christian Castillo, Claudio Olea-Azar, Mauricio Moncada-Basualto

**Affiliations:** 1 Department of Molecular Pharmacology and Clinical, Faculty of Medicine, University of Chile, Santiago 8380453, Chile; josue.pozo@uazuay.edu.ec; 2 Laboratorio de Química-Médica, Facultad de Ciencia y Tecnología, Universidad del Azuay, Av. 24 de Mayo 777, Cuenca 010204, Ecuador; 3 Instituto de Química Médica (CSIC), Juan de la Cierva 3, 28006 Madrid, Spain; uvejotaran@gmail.com; 4 Instituto Universitario de Investigación y Desarrollo Tecnológico, Universidad Tecnológica Metropolitana, Santiago 8940577, Chile; mzunigab@utem.cl (M.Z.-B.);; 5 Free Radical and Antioxidants Laboratory, Inorganic and Analytical Department, Faculty of Chemical and Pharmaceutical Sciences, University of Chile, Santiago 8380492, Chile; 6 Programa de Biología Integrativa, Instituto de Ciencias Biomédicas, Facultad de Medicina, Universidad de Chile, Santiago 8380453, Chileccastillor@uchile.cl (C.C.)

**Keywords:** trypanocidal activity, 5-Nitroindazolin-3-ones, chagas disease, oxidative stress, apoptosis induction

## Abstract

Chagas disease is a prevalent health problem in Latin America which has received insufficient attention worldwide. Current treatments for this disease, benznidazole and nifurtimox, have limited efficacy and may cause side effects. A recent study proposed investigating a wide range of nitroindazole and indazolone derivatives as feasible treatments. Therefore, it is proposed that adding a nitro group at the 5-position of the indazole and indazolone structure could enhance trypanocidal activity by inducing oxidative stress through activation of the nitro group by NTRs (nitroreductases). The study results indicate that the nitro group advances free radical production, as confirmed by several analyses. Compound **5a** (5-nitro-2-picolyl-indazolin-3-one) shows the most favorable trypanocidal activity (1.1 ± 0.3 µM in epimastigotes and 5.4 ± 1.0 µM in trypomastigotes), with a selectivity index superior to nifurtimox. Analysis of the mechanism of action indicated that the nitro group at the 5-position of the indazole ring induces the generation of reactive oxygen species (ROS), which causes apoptosis in the parasites. Computational docking studies reveal how the compounds interact with critical residues of the NTR and FMNH_2_ (flavin mononucleotide reduced) in the binding site, which is also present in active ligands. The lipophilicity of the studied series was shown to influence their activity, and the nitro group was found to play a crucial role in generating free radicals. Further investigations are needed of derivatives with comparable lipophilic characteristics and the location of the nitro group in different positions of the base structure.

## 1. Introduction

American trypanosomiasis, or Chagas disease, is produced by the parasite *Trypanosoma cruzi* (*T. cruzi*) and is endemic in 21 Latin American countries. However, due to global migration flows, it has also been found in other parts of the world, including the United States, Europe, Asia, and Africa [1]. Some estimates indicate that between 6 and 7 million people worldwide are infected with *T. cruzi* and 70 million people are at risk of contracting the disease. In addition, up to 12,000 people die each year from causes associated with the disease [2]. According to the World Health Organization (WHO), and like other neglected tropical diseases (NTDs), “Chagas disease is an indicator of poverty and disadvantage: it affects populations with little visibility and little political voice, causes stigma and discrimination, is relatively neglected by investigators and has a considerable impact on morbidity and mortality” [3].

Current chemotherapy for Chagas disease is restricted to only two nitroheterocyclic drugs: nifurtimox (NFX) and benznidazole (BNZ). These drugs are shown to be highly effective in acute, congenital, and early chronic (pediatric) cases, but their efficacy diminishes notably in the chronic phase of the disease [4]. In addition, long-term treatment with these drugs often leads to the emergence of severe side effects such as anorexia, psychic alterations, digestive manifestations, bone marrow depression, peripheral polyneuropathy, hypersensitivity, lymphadenopathy, thrombocytopenic purpura, and agranulocytosis [5,6,7]. Additionally, these drugs cannot be administered during pregnancy since they are teratogenic; this makes the development of new, more effective, and less toxic drugs necessary and urgent [8].

**Figure 1:**
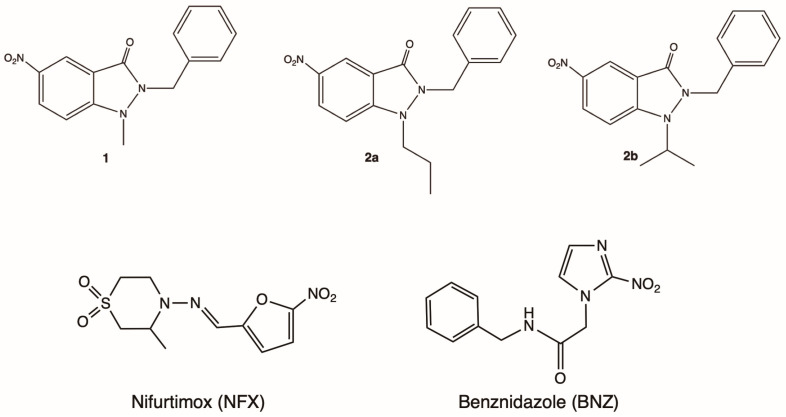
5-Nitroindazolin-3-ones with trypanocidal activity previously described [15,18] and structures of nifurtimox and benznidazole.

The NFX and BNZ (Figure 1) are prodrugs activated by *T. cruzi* nitroreductase (*Tc*NTR) [9,10]. *Tc*NTR promotes a two-electron serial reduction in the nitro group of the drugs, forming hydroxylamine via a nitroso intermediate which can be metabolized to a nitrenium ion and other highly toxic derivatives [11]. Therefore, activating nitro derivatives is critical to generating more efficient trypanocidal agents. Our research group has evaluated the trypanocidal activity of nitrocompounds 5-nitroindazoles and 6-nitroquinaxolines [12,13,14], whose mechanism of action was associated with the generation of oxidative stress on the epimastigotes and trypomastigotes forms of the Dm28c strain. Nitroindazole derivatives have received greater attention due to their physicochemical properties and observed trypanocidal activity, which is why they are considered a privileged structure for designing new trypanocidal agents.

It is known that 1,2-Disubstituted 5-nitroindazolin-3-ones exhibit notable trypanocidal properties, particularly compounds **1**, **2a**, and **2b** (Figure 1), which are effective against CL-B5 epimastigotes with low cytotoxicity [15]. They also show significant activity against intracellular amastigotes [16,17] and reduce parasitemia in mice [17]. 

Fonseca-Berzal et al. [18] reported similar activity for 5-nitroindazolin-3-ones with different aromatic substituents, with IC_50_ values between 1.04 and 6.17 µM. A correlation exists between trypanocidal activity and lipophilicity, where compounds with intermediate lipophilicity are more effective.

**Figure 2:**
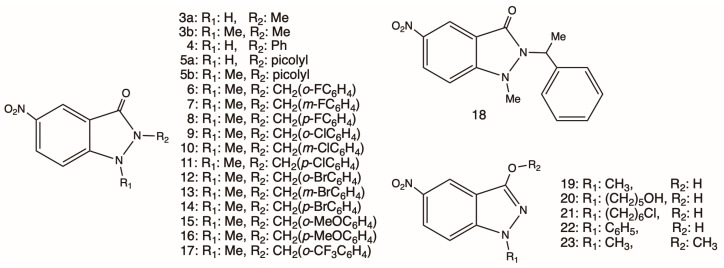
5-Nitroindazolin-3-ones and other 5-nitroindazole derivatives studied in this work.

In this work, we prepared and evaluated the trypanocidal activity against the epimastigote and trypomastigote forms of *T. cruzi* strain Dm28c of a series of 1,2-disubstituted 5-nitroindazolin-3-ones that contain at position 2 of the indazole ring benzyl groups with different substituents at positions 2, 3, and 4 (halogens, CF_3_, and OMe) and methyl group at position 1 (Figure 2). Other 5-nitroindazole derivatives were also prepared for comparison concerning the susceptibility of the strain under study.

Furthermore, the reduction mechanism of compounds was studied using cyclic voltammetry and electron spin resonance (ESR). Likewise, the interaction with tcNTR and its relationship with the generation of oxidative stress were evaluated as potential mechanisms of action.

## 2. Results and Discussion

### 2.1. Synthesis of 1,2-Disubstituted 5-nitroindazolin-3-ones

These compounds have been previously prepared by alkylation of 1-substituted indazol-3-ols with alkyl halides in the presence of a base (K_2_CO_3_, Cs_2_CO_3_, etc.), but in these cases, the isomeric *N*_1_,*O*-disubstituted derivatives are usually the major reaction products [15,18,19]. 

**Scheme 1:**
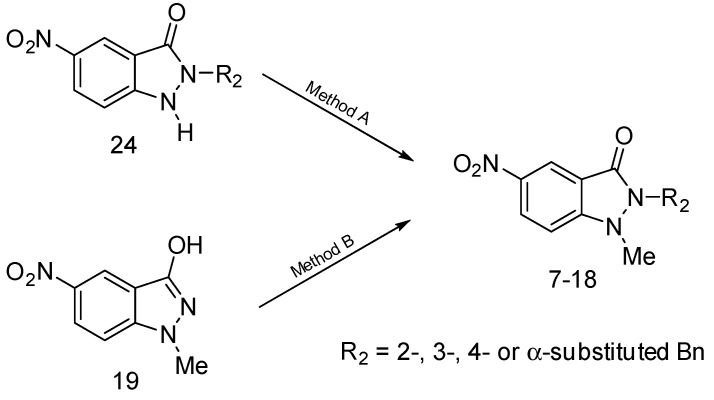
Synthesis of 5-nitroindazolin-3-ones **7**–**18**. Reagents and conditions: Method A, for **7**–**18**: MeI, K_2_CO_3_, DMF, RT, overnight, 95–98%. Method B, for **8**–**12**, **14**, and **17**: substituted benzyl bromide, DMF, 150 °C, 4 h, 89–96%.

On the other hand, 1,2-disubstituted indazolinones can also be prepared by alkylation of the corresponding 2-substituted derivatives (such as **24**, Scheme 1) with the required alkyl halides [19] in the presence of K_2_CO_3_; variable amounts of the isomeric 3-alkoxy-2-alkyl-2*H*-indazoles are also obtained in these processes [15,20,21], but the latter are very minor byproducts in the case of methylation reactions and this procedure has been used for the preparation of all the studied compounds (reported procedure [19] and the related method A). The main drawback of this method is, however, that the starting 2-substituted indazolin-3-ones **24**, prepared in turn by alkylation of 1-ethoxycarbonylindazol-3-ols followed by removal of the protecting COOEt group, are only obtained in moderate yield (<60%) owing to the concomitant production of the isomeric 3-alkoxyindazoles [19].

Additionally, 1-methyl-2-benzylindazolin-3-ones can be obtained in good yield (≥89%) by treatment of the easily available 1-methyl-5-nitroindazol-3-ol **19** with the corresponding benzyl bromides in DMF at 150 °C in the absence of a base [18]; an excess of the alkylating reagent is needed, owing to the competitive reaction of the latter with hot DMF to afford the corresponding *N*,*N*-dimethylbenzylamines and *N*-methyldibenzylamines [22]. This procedure has been used to prepare **8**–**12**, **14**, and **17** (method B). The NMR spectra of the compounds are shown in Supplementary Figures S1–S12.

2-(3-Fluorobenzyl)-1-methyl-5-nitro-1,2-dihydro-3*H*-indazol-3-one (**7**):

Yield: 0.87 g (96%) (method A). Mp 125–127 °C (2-PrOH). ^1^H NMR (300 MHz, DMSO-*d*_6_) δ 8.50 (d, *J* = 2.1 Hz, 1H, 4-H), 8.37 (dd, *J* = 9.3, 2.1 Hz, 1H, 6-H), 7.65 (d, *J* = 9.3 Hz, 1H, 7-H), 7.36 (m, 1H, Bn 5-H), 7.10 (m, 3H, Bn 2-H, 4-H, 6-H), 5.21 (s, 2H, CH_2_), 3.50 (s, 3H, CH_3_). ^13^C NMR (75 MHz, DMSO-*d*_6_) δ 162.13 (d, *J* = 242.8 Hz, Bn C-3), 160.32 (C-3), 149.61 (C-7a), 141.25 (C-5), 139.08 (d, *J* = 7.1 Hz, Bn C-1), 130.75 (d, *J* = 8.3 Hz, Bn C-5), 127.10 (C-6), 123.23 (d, *J* = 2.6 Hz, Bn C-6), 120.30 (C-4), 115.31 (C-3a), 114.59 (d, *J* = 20.7 Hz), 114.11 (d, *J* = 21.7 Hz) (Bn C-2, C-4), 111.96 (C-7), 44.10 (CH_2_), 35.21 (CH_3_). Anal. calcd for C_15_H_12_FN_3_O_3_ (301.28): C 59.80; H 4.01; N 13.95. Found: C 59.58; H 4.30; N 13.74.

2-(4-Fluorobenzyl)-1-methyl-5-nitro-1,2-dihydro-3*H*-indazol-3-one (**8**):

Yield: 0.88 g (97%) (method A); 1.44 g (95%) (method B). Mp 139–141 °C (2-PrOH). ^1^H NMR (300 MHz, DMSO-*d*_6_) δ 8.49 (d, *J* = 2.1 Hz, 1H, 4-H), 8.36 (dd, *J* = 9.0, 2.1 Hz, 1H, 6-H), 7.64 (d, *J* = 9.0 Hz, 1H, 7-H), 7.30 (m, 2H, Bn 2-H, 6-H), 7.14 (m, 2H, Bn 3-H, 5-H), 5.17 (s, 2H, CH_2_), 3.49 (s, 3H, CH_3_). ^13^C NMR (75 MHz, DMSO-*d*_6_) δ 161.62 (d, *J* = 242.2 Hz, Bn C-4), 160.31 (C-3), 149.60 (C-7a), 141.25 (C-5), 132.50 (d, *J* = 3.1 Hz, Bn C-1), 129.46 (d, *J* = 8.2 Hz, Bn C-2, C-6), 127.10 (C-6), 120.30 (C-4), 115.53 (d, *J* = 21.5 Hz, Bn C-3, C-5), 115.39 (C-3a), 111.96 (C-7), 43.90 (CH_2_), 35.23 (CH_3_). Anal. calcd for C_15_H_12_FN_3_O_3_ (301.28): C 59.80; H 4.01; N 13.95. Found: C 59.84; H 3.73; N 14.14.

2-(2-Chlorobenzyl)-1-methyl-5-nitro-1,2-dihydro-3*H*-indazol-3-one (**9**): 

Yield: 0.91 g (95%) (method A); 1.47 g (92%) (method B). Mp 179–181 °C (2-PrOH). ^1^H NMR (300 MHz, DMSO-*d*_6_) δ 8.51 (d, *J* = 2.4 Hz, 1H, 4-H), 8.39 (dd, *J* = 9.3, 2.4 Hz, 1H, 6-H), 7.69 (d, *J* = 9.3 Hz, 1H, 7-H), 7.49 (dd, *J* = 8.1, 1.8 Hz, 1H, Bn 3-H), 7.30 (m, 2H, Bn 4-H, 5-H), 7.03 (d, *J* = 7.2, 1H, Bn 6-H), 5.25 (s, 2H, CH_2_), 3.47 (s, 3H, CH_3_). ^13^C NMR (75 MHz, DMSO-*d*_6_) δ 160.19 (C-3), 149.71 (C-7a), 141.35 (C-5), 133.31 (Bn C-1), 131.63 (Bn C-2), 129.57, 129.49, 128.44, 127.63 (Bn C-3, C-4, C-5, C-6), 127.14 (C-6), 120.30 (C-4), 115.39 (C-3a), 112.14 (C-7), 42.63 (CH_2_), 35.38 (CH_3_). Anal. calcd for C_15_H_12_ClN_3_O_3_ (317.73): C 56.70; H 3.81; N 13.23. Found: C 56.55; H 4.09; N 13.33.

2-(3-Chlorobenzyl)-1-methyl-5-nitro-1,2-dihydro-3*H*-indazol-3-one (**10**):

Yield: 0.91 g (95%) (method A); 1.50 g (94%) (method B). Mp 154–156 °C (2-PrOH). ^1^H NMR (300 MHz, DMSO-*d*_6_) δ 8.50 (d, *J* = 2.1 Hz, 1H, 4-H), 8.37 (dd, *J* = 9.0, 2.1 Hz, 1H, 6-H), 7.65 (d, *J* = 9.0 Hz, 1H, 7-H), 7.35 (m, 3H, Bn 2-H, 4-H, 5-H), 7.20 (m, 1H, Bn 6-H), 5.20 (s, 2H, CH_2_), 3.50 (s, 3H, CH_3_). ^13^C NMR (75 MHz, DMSO-*d*_6_) δ 160.35 (C-3), 149.72 (C-7a), 141.31 (C-5), 138.78 (Bn C-1), 133.24 (Bn C-3), 130.63 (Bn C-2), 127.76 (C-6), 127.17, 127.14, 125.92 (Bn C-4, C-5, C-6), 120.31 (C-4), 115.36 (C-3a), 112.04 (C-7), 44.02 (CH_2_), 35.30 (CH_3_). Anal. calcd for C_15_H_12_ClN_3_O_3_ (317.73): C 56.70; H 3.81; N 13.23. Found: C 56.83; H 3.81; N 13.44.

2-(4-Chlorobenzyl)-1-methyl-5-nitro-1,2-dihydro-3*H*-indazol-3-one (**11**):

Yield: 0.92 g (97%) (method A); 1.48 g (93%) (method B). Mp 128–130 °C (2-PrOH). ^1^H NMR (300 MHz, DMSO-*d*_6_) δ 8.49 (d, *J* = 2.1 Hz, 1H, 4-H), 8.36 (dd, *J* = 9.3, 2.1 Hz, 1H, 6-H), 7.63 (d, *J* = 9.3 Hz, 1H, 7-H), 7.38 (d, *J* = 8.5 Hz, 2H, Bn 3-H, 5-H), 7.28 (d, *J* = 8.5 Hz, 2H, Bn 2-H, 6-H), 5.18 (s, 2H, CH_2_), 3.49 (s, 3H, CH_3_). ^13^C NMR (75 MHz, DMSO-*d*_6_) δ 160.28 (C-3), 149.57 (C-7a), 141.22 (C-5), 135.23 (Bn C-1), 132.38 (Bn C-4), 129.15 (Bn C-2, C-6), 128.64 (Bn C-3, C-5), 127.06 (C-6), 120.24 (C-4), 115.30 (C-3a), 111.91 (C-7), 43.94 (CH_2_), 35.19 (CH_3_). Anal. calcd for C_15_H_12_ClN_3_O_3_ (317.73): C 56.70; H 3.81; N 13.23. Found: C 56.48; H 4.10; N 13.13.

2-(2-Bromobenzyl)-1-methyl-5-nitro-1,2-dihydro-3*H*-indazol-3-one (**12**): 

Yield: 1.06 g (98%) (method A); 1.74 g (96%) (method B). Mp 182–184 °C (2-PrOH). ^1^H NMR (300 MHz, DMSO-*d*_6_) δ 8.52 (d, *J* = 2.2 Hz 1H, 4-H), 8.40 (dd, *J* = 9.3, 2.2 Hz, 1H, 6-H), 7.70 (d, *J* = 9.3 Hz, 1H, 7-H), 7.67 (d, *J* = 9.0 Hz, 1H, Bn 3-H), 7.27 (m, 2H, Bn 4-H, 5-H), 6.94 (d, *J* = 7.2 Hz, 1H, Bn 6-H), 5.21 (s, 2H, CH_2_), 3.49 (s, 3H, CH_3_). ^13^C NMR (75 MHz, DMSO-*d*_6_) δ 160.14 (C-3), 149.56 (C-7a), 141.34 (C-5), 134.85 (Bn C-1), 132.86 (Bn C-3), 129.73 (Bn C-4), 128.23, 128.16 (Bn C-5, C-6), 127.18 (C-6), 121.60 (Bn C-2), 120.36 (C-4), 115.34 (C-3a), 112.14 (C-7), 45.03 (CH_2_), 35.33 (CH_3_). Anal. calcd for C_15_H_12_BrN_3_O_3_ (362.18): C 49.74; H 3.34; N 11.60. Found: C 49.58; H 3.59; N 11.69.

2-(3-Bromobenzyl)-1-methyl-5-nitro-1,2-dihydro-3*H*-indazol-3-one (**13**):

Yield: 1.04 g (96%) (method A). Mp 149–151 °C (2-PrOH). ^1^H NMR (300 MHz, DMSO-*d*_6_) δ 8.50 (d, *J* = 2.3 Hz 1H, 4-H), 8.37 (dd, *J* = 9.1, 2.3 Hz, 1H, 6-H), 7.65 (d, *J* = 9.1 Hz, 1H, 7-H), 7.48 (m, 2H, Bn 2-H, 4-H), 7.25 (m, 2H, Bn 5-H, 6-H), 5.19 (s, 2H, CH_2_), 3.49 (s, 3H, CH_3_). ^13^C NMR (75 MHz, DMSO-*d*_6_) δ 160.35 (C-3), 149.74 (C-7a), 141.32 (C-5), 139.05 (Bn C-1), 130.94 (Bn C-4 or C-5), 130.69 (Bn C-2), 130.08 (Bn C-5 or C-4), 127.18 (C-6), 126.32 (Bn C-6), 121.85 (Bn C-3), 120.35 (C-4), 115.37 (C-3a), 112.07 (C-7), 43.96 (CH_2_), 35.33 (CH_3_). Anal. calcd for C_15_H_12_BrN_3_O_3_ (362.18): C 49.74; H 3.34; N 11.60. Found: C 49.79; H 3.21; N 11.39.

2-(4-Bromobenzyl)-1-methyl-5-nitro-1,2-dihydro-3*H*-indazol-3-one (**14**):

Yield: 1.06 g (98%) (method A); 1.71 g (94%) (method B). Mp 144–146 °C (2-PrOH). ^1^H NMR (300 MHz, DMSO-*d*_6_) δ 8.50 (d, *J* = 2.0 Hz, 1H, 4-H), 8.36 (dd, *J* = 9.3, 2.0 Hz, 1H, 6-H), 7.64 (d, *J* = 9.3 Hz, 1H, 7-H), 7.52 (d, *J* = 8.1 Hz, 2H, Bn 3-H, 5-H), 7.21 (d, *J* = 8.1 Hz, 2H, Bn 2-H, 6-H), 5.16 (s, 2H, CH_2_), 3.49 (s, 3H, CH_3_). ^13^C NMR (75 MHz, DMSO-*d*_6_) δ 160.30 (C-3), 149.59 (C-7a), 141.24 (C-5), 135.68 (Bn C-1), 131.59 (Bn C-3, C-5), 129.51 (Bn C-2, C-6), 127.11 (C-6), 120.93 (Bn C-4), 120.28 (C-4), 115.31 (C-3a), 111.96 (C-7), 44.00 (CH_2_), 35.21 (CH_3_). Anal. calcd for C_15_H_12_BrN_3_O_3_ (362.18): C 49.74; H 3.34; N 11.60. Found: C 49.98; H 3.49; N 11.37.

2-(2-Methoxybenzyl)-1-methyl-5-nitro-1,2-dihydro-3*H*-indazol-3-one (**15**):

Yield: 0.90 g (96%) (method A). Mp 210–212 °C (2-PrOH). ^1^H NMR (300 MHz, DMSO-*d*_6_) δ 8.50 (d, *J* = 2.4 Hz, 1H, 4-H), 8.36 (dd, *J* = 9.0, 2.4 Hz, 1H, 6-H), 7.64 (d, *J* = 9.0 Hz, 1H, 7-H), 7.26 (m, *J* = 7.8, 7.8, 1.8 Hz, 1H, Bn 4-H), 7.01 (d, *J* = 7.8 Hz, 1H, Bn 3-H), 6.94 (dd, *J* = 7.8, 1.8 Hz, 1H, Bn 6-H), 6.85 (dd, *J* = 7.8, 7.8 Hz, 1H, Bn 5-H), 5.12 (s, 2H, CH_2_), 3.79 (s, 3H, OCH_3_), 3.47 (s, 3H, NCH_3_). ^13^C NMR (75 MHz, DMSO-*d*_6_) δ 160.22 (C-3), 156.54 (Bn C-2), 149.27 (C-7a), 141.10 (C-5), 129.13 (Bn C-4), 127.93 (Bn C-6), 126.94 (C-6), 123.68 (Bn C-1), 120.50 (Bn C-5), 120.24 (C-4), 115.37 (C-3a), 111.82 (C-7), 111.01 (Bn C-3), 55.46 (OCH_3_), 39.99 (CH_2_), 34.94 (NCH_3_). Anal. calcd for C_16_H_15_N_3_O_4_ (313.31): C 61.34; H 4.83; N 13.41. Found: C 61.23; H 4.97; N 13.49.

2-(4-Methoxybenzyl)-1-methyl-5-nitro-1,2-dihydro-3*H*-indazol-3-one (**16**):

Yield: 0.87 g (93%) (method A). Mp 139–141 °C (2-PrOH). ^1^H NMR (300 MHz, DMSO-*d*_6_) δ 8.48 (d, *J* = 2.1 Hz, 1H, 4-H), 8.34 (dd, *J* = 9.3, 2.1 Hz, 1H, 6-H), 7.61 (d, *J* = 9.3 Hz, 1H, 7-H), 7.20 (d, *J* = 8.6 Hz, 2H, Bn 2-H, 6-H), 6.86 (d, *J* = 8.6 Hz, 2H, Bn 3-H, 5-H), 5.10 (s, 2H, CH_2_), 3.69 (s, 3H, OCH_3_), 3.50 (s, 3H, NCH_3_). ^13^C NMR (75 MHz, DMSO-*d*_6_) δ 160.22 (C-3), 158.78 (Bn C-4), 149.35 (C-7a), 141.08 (C-5), 128.72 (Bn C-2, C-6), 128.14 (Bn C-1), 126.96 (C-6), 120.24 (C-4), 115.30 (C-3a), 114.04 (Bn C-3, C-5), 111.74 (C-7), 54.98 (OCH_3_), 44.09 (CH_2_), 35.08 (NCH_3_). Anal. calcd for C_16_H_15_N_3_O_4_ (313.31): C 61.34; H 4.83; N 13.41. Found: C 61.04; H 4.67; N 13.54.

1-Methyl-5-nitro-2-[2-(trifluoromethyl)benzyl]-1,2-dihydro-3*H*-indazol-3-one (**17**): 

Yield: 0.99 g (94%) (method A); 1.57 g (89%) (method B). Mp 211–213 °C (2-PrOH). ^1^H NMR (300 MHz, DMSO-*d*_6_) δ 8.56 (d, *J* = 2.1 Hz, 1H, 4-H), 8.43 (dd, *J* = 9.3, 2.1 Hz, 1H, 6-H), 7.82 (d, *J* = 7.5, 1H, Bn 3-H), 7.73 (d, *J* = 9.3 Hz, 1H, 7-H), 7.60 (dd, *J* = 7.5, 7.5 Hz, 1H, Bn 5-H), 7.52 (dd, *J* = 7.5, 7.5 Hz, 1H, Bn 4-H), 6.98 (d, *J* = 7.5 Hz, 1H, Bn 6-H), 5.34 (s, 2H, CH_2_), 3.45 (s, 3H, CH_3_). ^13^C NMR (75 MHz, DMSO-*d*_6_) δ 160.24 (C-3), 149.34 (C-7a), 141.36 (C-5), 134.10 (Bn C-1), 133.23 (Bn C-5), 128.21 (Bn C-4), 127.25 (C-6, Bn C-6), 126.29 (q, *J* = 6.2 Hz, Bn C-3), 125.89 (q, *J* = 30.0 Hz, Bn C-2), 124.30 (q, *J* = 272.5 Hz, CF_3_), 120.45 (C-4), 115.09 (C-3a), 112.12 (C-7), 41.47 (CH_2_), 35.00 (CH_3_). Anal. calcd for C_16_H_12_F_3_N_3_O_3_ (351.29): C 54.71; H 3.44; N 11.96. Found: C 54.87; H 3.73; N 11.87.

1-Methyl-5-nitro-2-(1-phenylethyl)-1,2-dihydro-3*H*-indazol-3-one (**18**): 

Yield: 0.87 g (98%) (method A). Mp 155–157 °C (2-PrOH). ^1^H NMR (300 MHz, DMSO-*d*_6_) δ 8.44 (d, *J* = 2.1 Hz, 1H, 4-H), 8.35 (dd, *J* = 9.3, 2.1 Hz, 1H, 6-H), 7.62 (d, *J* = 9.3 Hz, 1H, 7-H), 7.42–7.23 (m, 5H, PhH), 5.75 (q, *J* = 7.2 Hz, 1H, CH), 3.33 (s, 3H, NCH_3_), 1.85 (d, *J* = 7.2 Hz, 3H, CCH_3_). ^13^C NMR (75 MHz, DMSO-*d*_6_) δ 161.18 (C-3), 150.46 (C-7a), 141.36 (C-5), 140.08 (Ph C-1), 128.50 (Ph C-3, C-5), 127.53, 127.16 (C-6, Ph C-4), 126.55 (Ph C-2, C-6), 120.13 (C-4), 115.96 (C-3a), 112.13 (C-7), 52.88 (CH), 37.15 (NCH_3_), 17.60 (CCH_3_). Anal. calcd for C_16_H_15_N_3_O_3_ (297.31): C 64.64; H 5.09; N 14.13. Found: C 64.74; H 5.03; N 14.34.

### 2.2. Cyclic Voltammetry

**Figure 3:**
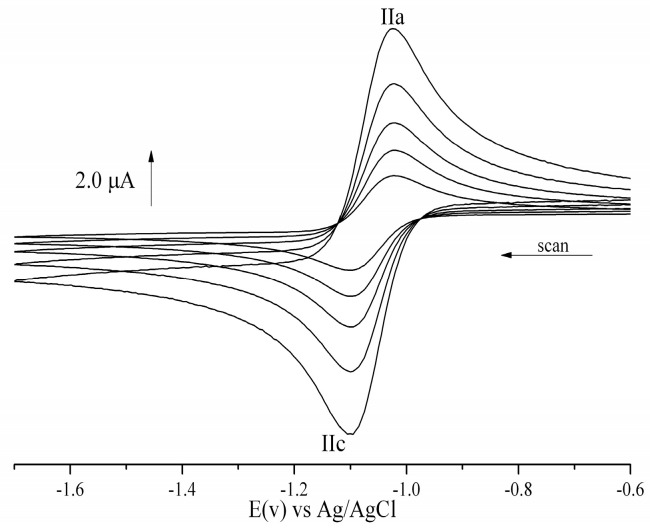
Cyclic voltammogram of compound **3b**, recorded at different scan rates between 0.1 and 2.0 V/s.

The 5-nitroindazolin-3-ones without labile protons in their structure (**3b**, **5b**, **6**–**18**) presented a similar electrochemical behavior. Figure 3 shows a voltammogram with a single reversible couple corresponding to the formation of the nitroanion radical. The 5-nitroindazolin-3-ones without labile protons presented less negative reduction potential than acidic protons derivatives (Table 1). Furthermore, no significant influence of the chemical nature and position of the substituents in the benzyl fraction could be evidenced. On the other hand, a similar electrochemical behavior was found for compound **23** corresponding to 5-nitroindazole, but with a more negative reduction potential, possibly due to the loss of the electro-attracting effect of the carbonyl in position 3. The reduction potentials for this couple were similar to those described by Olea-Azar et al. [23] for a series of 3-benzyloxy-5-nitroindazole derivatives, as described by Rodriguez et al. [24] for a series of 5-nitroindazole with 3-Alcoxy, 3-hydroxy and 3-oxo substituents, and Folch-Cano et al. [12] for a 1,2-disubstituted 5-nitroindazolin-3-ones and 2-substituted 3-alkoxy-5-nitro-2*H*-indazoles.

**Figure 4:**
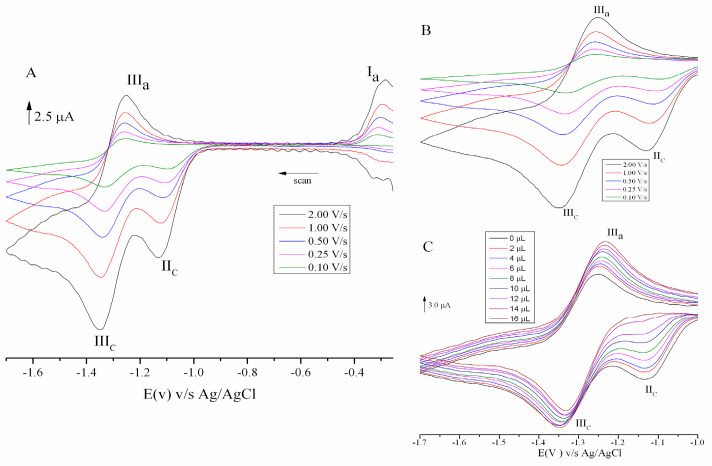
(**A**) Cyclic voltammogram of compound **19** with a potential sweep between −2.0 and 0.0 V and sweep speeds between 0.1 and 2.0 V/s. (**B**) Cyclic voltammogram using a potential range between −1.8 and −0.9 V and sweep speeds between 0.1 and 2.0 V/s. (**C**) Cyclic voltammogram using a speed of 2.0 V/s: (i) the first black line without NaOH; (ii) the red line in the presence of 30 mM NaOH.

The 5-nitroindazoles presented a similar electrochemical behavior (except compound **23**). Figure 4A shows a cathodic peak assigned as IIc, a couple IIIc/IIIa, and an isolated oxidation signal (Ia). The irreversible cathodic peak IIc close to −1.1 V is attributed to the reduction of an acid species that disappears when the medium is alkalized, as shown in Figure 4C. This self-protonation process corresponds to an acid–base equilibrium in aprotic media, a typical behavior shown by nitro compounds with acidic residues in their structure [23,25,26]. Peak IIIc was assigned to the electrochemical reduction of nitro anion to the corresponding radical, which is oxidized in a quasi-reversible process that generates couple IIIc/IIIa (Figure 4B), according to [27,28]. The peak Ia is attributed to the oxidation of the hydroxylamine derivative generated by a corresponding nitroso compound.

The 5-nitroindazolinones with labile protons in their structure (**3a**, **4**, and **5a**) presented a similar electrochemical behavior. However, the voltammograms did not show the anodic signal attributed to the oxidation of hydroxylamine. The latter is attributed to the fact that the labile protons have lower acidity since they can be retained with greater force due to the attracting effect of the carbonyl group in position 3. Additionally, the dependence of cathodic current intensity (Ipc) on sweep speed was evaluated, finding a linear relationship with a slope close to 0.5, which, according to Robledo-O’Ryan et al. (Robledo-O’Ryan et al., 2017) [29], would obey a diffusional type of control towards the surface of the electrode.

Table 1 shows the reduction potentials of all compounds. The 5-nitroindazolones presented the least negative reduction potentials, which is most likely the presence of the carbonyl group in the 3-position of their basic structure. When comparing the Epc values of the 5-nitroindazole derivatives, it is evident that compound **22** has the smallest negative value; This means that it is more easily reducible and has a better capacity to generate radical species. However, the nitro compounds NFX and BNZ presented less negative medium wave potentials (E_1/2_) than those under study. This demonstrates the difficulty of these derivatives in forming the nitro radical that could generate oxidative stress in the parasite *T. cruzi*.

On the other hand, 5-nitroindazolinones without labile protons presented reduction potentials similar to the drug metronidazole, which could be biotransformed by nitroreductases, as described by Livertoux et al. [30]. From electrochemical studies, it is postulated that 5-nitroindazolinones could present the highest trypanocidal activity of the series under study since the less negative reduction potentials would give indications about the capacity to generate oxidative stress in *T. cruzi* parasites, which is described for this type of compound [13].

#### Characterization of Radicals by ESR

To obtain more information about the reduction mechanism of 5-nitroindazole and 5-nitroindazolin-3-one derivatives, the ESR spectra of radicals generated by electrolysis in situ were recorded under identical conditions as those used in CV studies, applying the potentials of reduction determined. A semi-empirical simulation was used with WinSIM 9 software to determine the hyperfine coupling constants [31].

**Figure 5:**
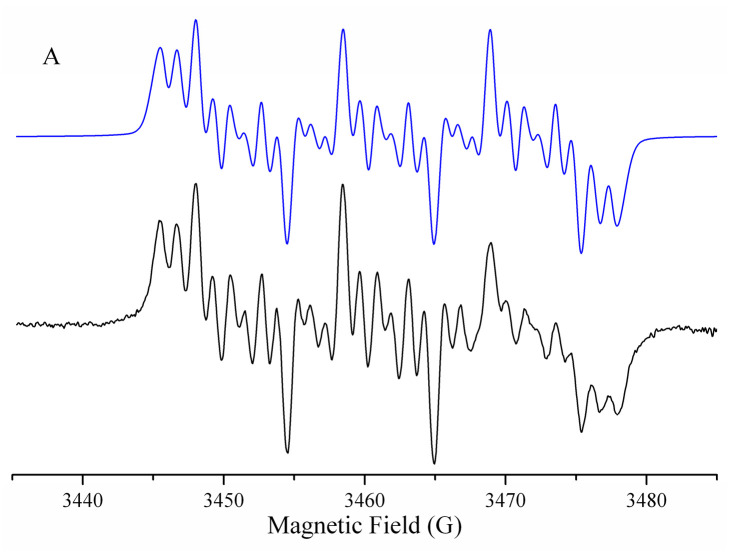

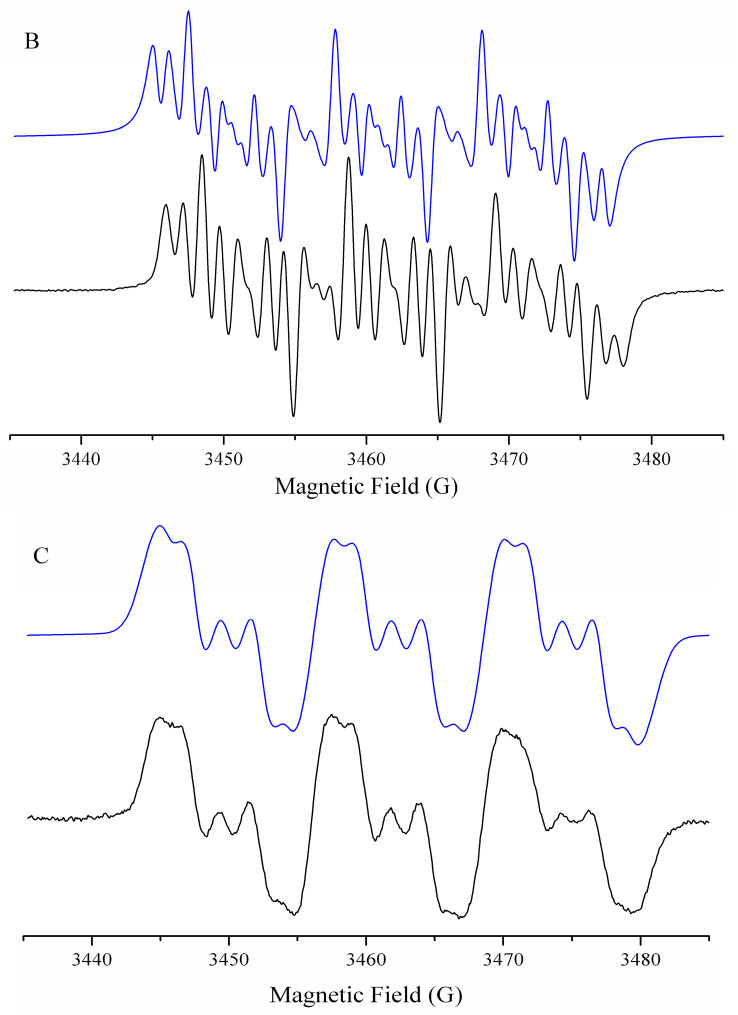
Experimental (black) and WimSIM (blue) simulated the ESR spectrum of (**A**) compound **5a**, (**B**) compound **10**, and (**C**) compound **23** at room temperature in DMSO.

The simulation of the spectra obtained for 5-nitroindazolin-3-one was similar for all compounds with or without labile protons (**1**–**18**). Figure 5A,B shows a hyperfine pattern of 29 lines attributable to two triplets attributed to the nitrogen group of the nitro group (N_1_) and to one of the ring nitrogens of 5-nitroindazolin-3-one (N_2_), three doublets of the hydrogens of the indazole ring (H_1_, H_2_, and H_3_). The obtained coupling constants would indicate that the N_2_-linked substituent would act as an electron donor since it prevents the delocalization of the unpaired electron in the aromatic substituent of compounds **4**–**17**. Therefore, the spin density distribution would be more delocalized near the nitro group (Table 2).

The ESR spectra obtained for the series of 5-nitroindazoles were similar for all compounds. Figure 5C shows a similar spectrum for compounds **19**–**23**, in which fifteen signals were assigned: one triplet assigned to the nitrogen of the nitro group (N_1_) three doublets attributed to the interaction of the unpaired electron with the nuclei of the hydrogens of the aromatic system (H_1_, H_2_, and H_3_). This spectrum was similar to that obtained by Rodriguez et al. for a 3-hydroxy-5-nitroindazole derivative [24]. For all compounds, the coupling constants indicated an excellent localization in a nitro group with less delocalization in the pyrazole ring.

Finally, it is possible to postulate that the reversible reduction mechanism for both compounds involves forming a radical nitro anion whose delocalization of unpaired electrons is mainly centered on the benzene ring. 

The reduction potentials of compounds make it possible to postulate the formation of nitroanion radicals using cellular reductases and their redox recycling as a possible mechanism of trypanocidal action through oxidative stress.

**Scheme 2:**
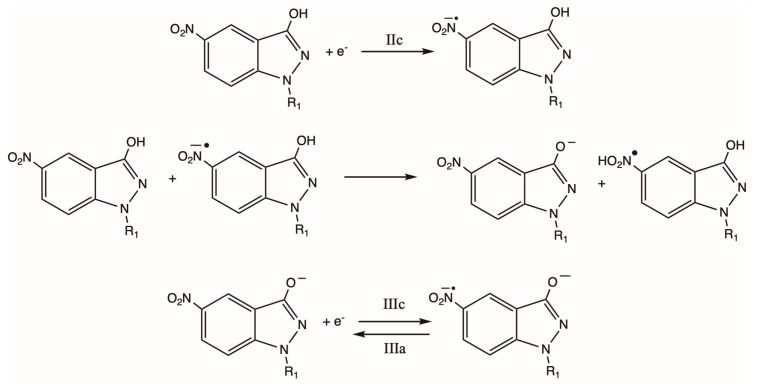
Proposed reduction mechanism for both 5-nitroindazole and 5-nitroindazolinone series with labile protons.

Based on the results, the following reduction mechanism was postulated for 5-nitroindazole derivatives with labile protons (Scheme 2).

### 2.3. Determination of Cytotoxic Activity against T. cruzi (Dm28c) Epimastigotes and Trypomastigote Forms

The trypanocidal activity of both series of compounds was evaluated against the epimastigote and trypomastigote forms of *T. cruzi*; it was found that both series presented trypanocidal activity and that they were more active in the non-infective form of the parasite. The series of 5-nitroindazolones had higher trypanocidal activity, with compounds **3a** and **5a** more active than NFX in both cell forms. In Table 3, the selectivity index of compound **5a** was higher than that of nifurtimox; this would indicate that despite its high cytotoxicity in mammalian cells, it is even more selective than the drug for clinical use. 

As for derivatives of 5-nitroindazolones, there is no connection between the lipophilicity of the compounds and their trypanocidal activity. It is postulated that the presence of the nitro group could generate oxidative stress as an antiparasitic mechanism of action, considering that the parasite has fewer endogenous antioxidant mechanisms than mammalian cells [29].

Furthermore, a heterocyclic substituent (**5a**) modulates the trypanocidal activity. The heteroatom enables interaction with enzymes, cell membranes, and other targets through non-covalent interactions. Fonseca-Berzal et al. [18] studied a series of 5-nitroindazole derivatives, where the compound with a 2-picolyl substituent showed considerable activity against the epimastigote and intracellular amastigote forms of *T. cruzi*, corroborating the results obtained for the series under study. Additionally, the absence of an R1 substituent improves the trypanocidal activity against epimastigotes.

For the 5-nitroindazole derivatives, it was found that the compounds were more active against the epimastigote form of *T. cruzi*. Compound **22** was the most active in this series. The latter may be related to the hydroxyl group in position 3, which could quench the radical species generated through the HAT mechanism and decrease the associated biological activity. The lipophilicity of the compounds does not influence their activity. As a substituent in R1 (**22**), the benzene ring increases the trypanocidal activity in the series under study, potentially due to interactions with various parasitic targets through van der Waals interactions.

Likewise, it was found for 5-nitroindazolin-3-ones that there is no relationship between the determined reduction potentials and trypanocidal activity, which suggests that the activation of the compounds by nitroreductases may not be directly related to trypanocidal activity since it is different from what was described by Gerpe et al. for a series of indazole N-oxide derivatives [32]. The latter differs for 5-nitroindazole derivatives where the most active compound also presented the least negative reduction potential. On the other hand, the trypanocidal activity of both series for this strain was similar to that described by Rodríguez et al. for a series of 5-nitroindazoles in epimastigote form in the Brener clone strain [33].

Regarding toxicity to mammalian cells, it was determined that the 5-nitroindazolone series is more toxic than the 5-nitroindazole series, which could be attributed to the presence of the keto group at position 3. Studies on the antileishmanial activity of 5-nitroindazole derivatives by Mollineda-Diogo et al. [34] showed similar cytotoxicity to the series under study in macrophage cells. Additionally, the presence of electron-withdrawing groups generally increases the cytotoxicity of molecules [18].

### 2.4. Action Mechanism

The activity of nitro compounds may be related to different mechanisms of trypanocidal action. A necrotic death of the parasites would trigger an exacerbated immune response and an inflammatory reaction that could damage the host’s infected tissues. For this reason, it is interesting that new treatments induce Programmed Cell Death (PCD) by apoptosis, in which these inflammatory problems do not develop [35].

Therefore, the potential mechanisms of action associated with characteristic events of apoptotic death were studied. Among the events studied are the permeability of the plasma membrane, the decrease in the level of cellular ATP, the alteration of the mitochondrial membrane potential, and the generation of reactive oxygen species and free radicals, all characteristics of apoptotic cell death [36,37].

Furthermore, we used molecular docking to evaluate the compounds’ interaction with nitroreductases and determine the importance of nitro radical generation in the observed trypanocidal activity.

#### 2.4.1. Plasma Membrane Permeability

**Figure 6:**
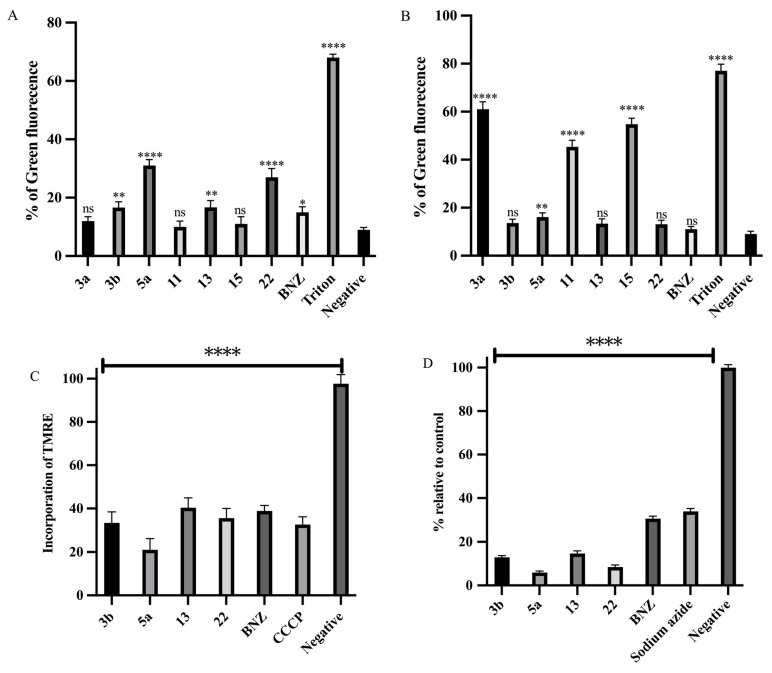
(**A**). Percentage of SYTOX Green probe incorporation of the most active compounds on *T. cruzi* trypomastigotes. (**B**). Percentage of SYTOX Green probe incorporation of the most active compounds on RAW 264.7 cells. (**C**). Percentage of TMRM probe incorporation by variation of the mitochondrial membrane potential on *T. cruzi* trypomastigote. (**D**). Percentage of ATP levels in *T. cruzi* trypomastigotes by the effect of the compounds with the highest trypanocidal activity. The significant difference compared to the control (one-way ANOVA with Dunnett post-test, ****: *p* ≤ 0.001; **: *p* ≤ 0.05; *: *p* ≤ 0.1). ns: non significative variation.

The permeability to the plasma membrane of the compounds with the highest selectivity index in the trypomastigote form of *T. cruzi* was investigated using the SYTOX Green fluorescent probe. Triton X-100 was used as a positive control. 5-nitroindazolin-3-ones (**3b**, **5a**, and **13**) and 5-nitroindazole (**22**) were found to alter plasma membrane permeability, unlike the trypanocidal drug BNZ (Figure 6A). The observed alteration was consistent with the previously determined trypanocidal activity, indicating a possible mechanism of action associated with apoptosis.

The plasma membrane regulates nutrient transport, pH homeostasis, and other ions and is essential for survival. Crossing a drug across the membrane can cause a perturbation in the plasma membrane potential and alter the flow of ions [38]. Therefore, not only compounds that alter the permeability of the plasma membrane can have an essential effect on homeostasis in trypanosomatids but also those that decrease its electrical potential, which would be interesting to evaluate for potential drugs with higher selectivity indices.

Additionally, the effect of the most cytotoxic compounds on the plasma membrane permeability of RAW 264.7 cells (**3a**, **11**, and **15**) was determined. Figure 6B shows that the most cytotoxic compounds significantly affect permeability, partly explaining their high cytotoxicity, unlike the most selective compounds. From a structural point of view, no influence of lipophilicity on the permeability of the parasite membrane was evident, so biological activity is not controlled by lipophilicity as a fundamental parameter of activity.

#### 2.4.2. Mitochondrial Membrane Potential (ψm)

In trypanosomatidae, unlike mammalian cells, the mitochondria are a single organelle that plays a crucial role in energy production. For this reason, it is considered an important therapeutic target. The effect of the most active compounds (**3b**, **5a**, **13**, and **22**) on the mitochondrial membrane potential (ψm) was determined by incorporating TMRE. The compounds showed a statistically significant impact compared to the negative control, with an activity order of **5a** > **3b**~**22** > **13**. This effect was comparable to that observed with CCCP and the drug BNZ (Figure 6C).

These results suggest that the mechanism of action of 5-nitroindazolin-3-one derivatives involves mitochondrial dysfunction associated with the generation of oxidative stress. Furthermore, it could result from blocking the electrophoretic accumulation of calcium by mitochondria, which could initially enhance the proton motive force generated by the electron transport chain in the inner mitochondrial membrane. This effect was followed by depolarization of the mitochondrial membrane, which could be attributed to the reduction in calcium levels and the consequent decrease in the activity of metabolic enzymes. Likewise, this blockage could have affected the function of acidocalcisomes, organelles specialized in accumulating protons, calcium, and short/long-chain polyphosphates. These acidic organelles are the main calcium reservoirs in trypanosomatids and are essential for the parasite’s survival [38,39].

Compound **5a**, with an intermediate polarity, exhibited the highest trypanocidal activity and the most significant effect on mitochondrial function. Therefore, it is postulated that the mechanism of action evaluated is associated with membrane-permeable compounds; the permeability and bioavailability of the compounds were more significant than those described by [40] for a series of 3-arylcoumarin derivatives, whose mechanism of action also involved the generation of oxidative stress in *T. cruzi* trypomastigotes. 5-Nitroindazolin-3-one derivatives are more active, confirming the postulated mechanism of action.

#### 2.4.3. Analysis of ATP Levels

Mitochondrial membrane potential is crucial to maintaining the electrochemical gradient in the respiratory chain, thus ensuring ATP generation and proper physiological function. The lethal effect of various trypanocidal compounds is attributed to the bioenergetic impairment in *T. cruzi*, which constitutes the focus in the development of these compounds to improve the treatment of Chagas disease [41].

ATP levels were determined in the trypomastigote form of *T. cruzi* for the most active compounds (**3b**, **5a**, **13**, and **22**), expressing them as a percentage concerning the negative control. As shown in Figure 6D, all compounds significantly reduced ATP levels, correlating with their trypanocidal activity. The decrease in ATP levels suggests cell death, possibly related to the production of reactive oxygen species caused by the activation of the nitro group of the compounds. This finding indicates a potential mechanism of action for these compounds, although not necessarily through apoptosis.

#### 2.4.4. Intracellular ROS Generation

**Figure 7:**
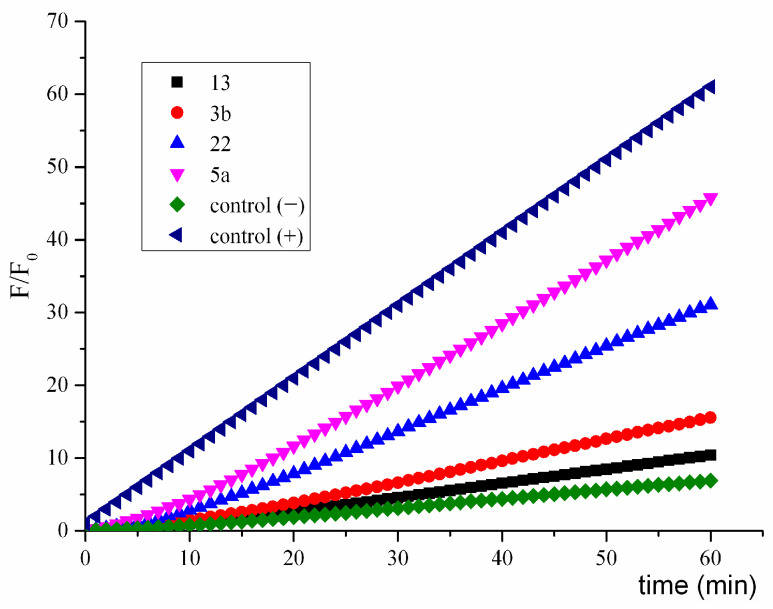
Increase in fluorescence as a function of time by generation of intracellular ROS in trypomastigotes of *T. cruzi*.

Intracellular reactive oxygen species were determined using dichlorofluorescein (DCF) probe quantification. All compounds tested increased fluorescence intensity (Figure 7). Compound **5a** presented the most significant increase in fluorescence intensity, related to the increase in the concentration of intracellular reactive species. This effect was more important than that found for BNZ and followed the same trend as the impact on mitochondrial function. However, determining intracellular ROS is a simpler and cheaper measure to develop than determining the variation of the mitochondrial membrane potential. Furthermore, this technique allows the simultaneous detection of permeability and ROS generation, being the screening technique to advance to more sophisticated evaluation systems for evaluating this mechanism of action.

Based on the results obtained, it can be postulated that the structure of 5-nitroindazolin-3-one **5a** would be a basic skeleton for the design of new trypanocidal compounds. These compounds must have intermediate lipophilicity, integrating electron acceptors into the aromatic system of the substituent to reduce reduction potentials and allow activation by cellular nitroreductases.

#### 2.4.5. Study of the Generation of Radical Species by Spin Trapping

To characterize the reactive radical species formed and confirm oxidative stress as a mechanism of action, the spin trap technique, using DMPO as a spin trap, was decided to be used on the trypomastigote form of *T. cruzi* in the presence of compound **5a**.

**Figure 8:**
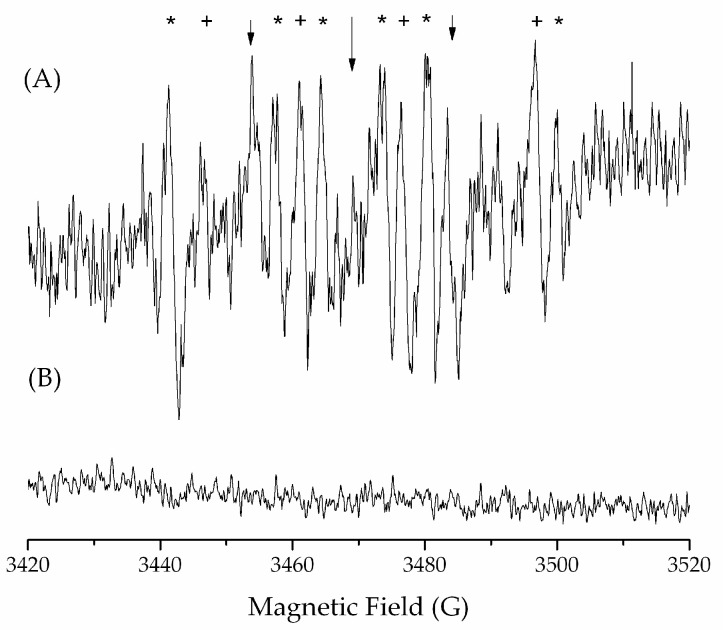
Spectra of the spin adducts generated in trypomastigote forms of *T. cruzi* (Dm28c) at room temperature. (**A**) Spectrum recorded with trypomastigotes incubated with compound **5a** and DMPO, marked with (*), (↓), and (+), radicals centered on carbon, DMPOOX, and hydroxyl radical, respectively. (**B**) Spectrum recorded in trypomastigotes with the DMPO spin trap.

Figure 8 shows that incubation with **5a** in the presence of NADPH under aerobic conditions produces a triplet (aN~15.5 G) corresponding to a DMPO-derived oxidized paramagnetic compound (DMPOX, 5,5-dimethyl-2-oxopyrroline-1-oxyl), marked with (↓) in the spectrum. In addition, a sextet corresponding to the trapping of a carbon-centered radical was generated (aN~16.00G aH~23.00 G, marked with *). In addition, a signal corresponding to the generation of the hydroxyl radical was found (aN = aH~14.00G, marked with +), as described by [13,42].

According to the results obtained, it is postulated that a possible mechanism of trypanocidal action involves the formation of free radicals due to the bioreduction of the nitro compounds under study. Therefore, a viable strategy for generating more robust and less cytotoxic trypanocidal drugs is to maintain the basic structure of 5-nitroindazolone by varying the lipophilic substituents in positions 1 and 2. Additionally, it is possible to form biomaterials that allow the controlled release of these compounds and reduce their cytotoxic effects on mammalian cells to improve the bioavailability of these compounds.

Two parameters were determined to obtain more information about the mechanism of oxidative stress associated with mitochondrial function: variation of the mitochondrial membrane potential and generation of intracellular ROS in trypomastigotes of *T. cruzi*.

#### 2.4.6. Interaction of Nitroreductase Receptor on *T. cruzi* and Compounds

**Figure 9:**
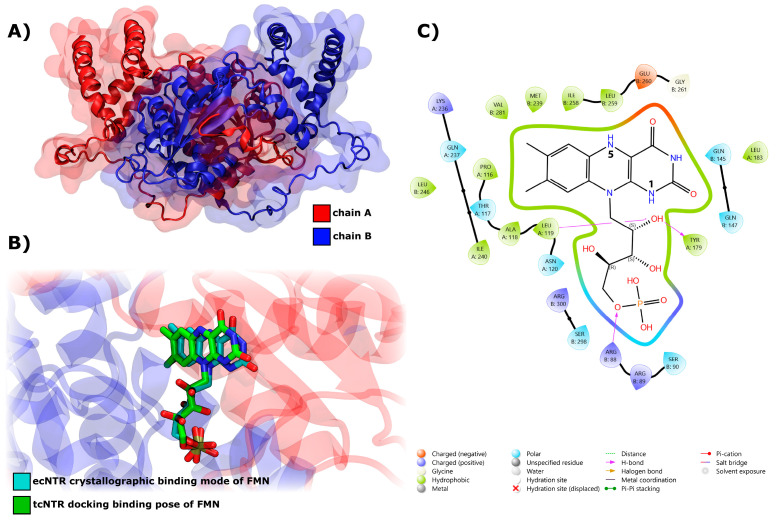
(**A**) Structure of modeled *Tc*NTR receptor including two monomers (chain A in red and chain B in blue, respectively). (**B**) Superposition of FMN binding mode into ecNTR receptor and *Tc*NTR docking result. (**C**) Ligand interaction diagram of FMNH_2_ and *Tc*NTR binding site.

Docking simulations were carried out for compounds **1** to **23** and Nifurtimox (NFX) in the *T. cruzi* nitroreductase receptor (*Tc*NTR) binding site. Figure 9A shows the modeled structure of the receptor obtained by the ESMFold v1.0 software (https://github.com/facebookresearch/esm, accessed on 14 October 2024). Our docking protocol showed a similar compulsory mode of FMN in the *Tc*NTR compared to the crystallographic structure of *E. coli* Nitroreductase (*ec*NTR) [43] and computational studies reported previously by [11], as displayed in Figure 9B. In detail, the interaction of docked FMN into the *Tc*NTR binding site is mainly regulated by hydrogen bonds between the ligand and residues A:L119, A:Y179, and B:R88 (Figure 9C). The output of this complex was used as the input for the docking simulations of compounds and Nifurtimox (NFX) into the binding site including the reduced form of FMN.

Table 4 compares the docking score values obtained for each compound and NFX active ligand. For synthesized compounds (**1**–**23**), a good agreement was observed within experimental and previously reported results, where active compounds **2a** and **2b** and our compounds **5a**, **7**, **1**, and **5b** showed the best docking scores. On the other hand, compounds **15**, **16**, **19**, and **23** showed lower values, which could explain their reduced trypanocidal activity.

**Figure 10:**
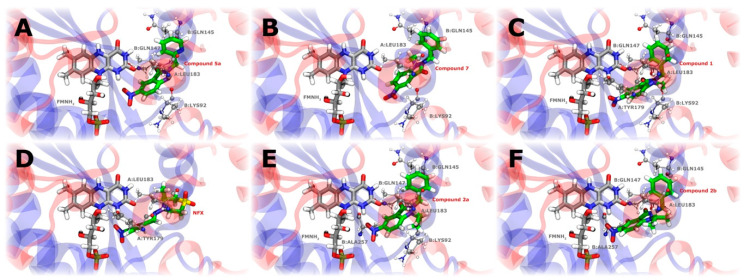
Ligand interaction maps of (**A**) **5a**, (**B**) **7**, (**C**) **1**, and active ligands (**D**) NFX, (**E**) **2a,** and (**F**) **2b** into the *Tc*NTR binding site predicted through docking calculations (carbons are in green). FMNH_2_ is depicted in the figure with gray carbons, while chains A and B of *Tc*NTR are colored red and blue, respectively.

The interaction modes of compounds **5a**, **7**, and **1** and active ligands **NFX**, **2a**, and **2b** in the binding site of the *Tc*NTR are specified in Figure 10. The docking results indicate that the nitro groups of the compounds are positioned at close distances to the N1 hydrogen (D_Nitro-N1_) of the reduced form of FMNH_2_, suggesting a potential interaction site. This proximity could facilitate an effective electron transfer from the reduced flavin cofactor, initiating the reduction of the nitro groups. Compound **5a** establishes multiple interactions within the binding site, forming a hydrogen bond with residue B:Q147 and hydrophobic interactions with residues A:L183, B:K92, and B:Q145. The hydrogen bond observed between compound **5a** and residue B-Q147 is mediated by the carbonyl oxygen of the compound interacting with the amide hydrogen of the glutamine side chain. The picolyl group of compound 5 likely facilitates these hydrophobic interactions, stabilizing the compound within the binding pocket. The docking score for compound **5a** is −7.3 kcal/mol, and it displayed a D_Nitro-N1_ distance of 2.36 Å. Similarly, compound **1** also forms a hydrogen bond with B:Q147 (involving the same atoms as in compound **5a**), while participating in hydrophobic interactions with A:Y179, A:L183, B:K92, and B:Q145. The presence of a phenyl ring adjacent to the carbonyl group contributes to these hydrophobic contacts. The docking score for compound **1** is −6.6 kcal/mol, with a D_Nitro-N1_ distance of 3.01 Å. In contrast, compound **7** does not form any hydrogen bonds but exhibits hydrophobic interactions with residues A:F32, A:L183, B:K92, and B:Q145. The presence of a methyl group (R_1_ = Me) and a meta-fluorophenylmethyl group (R_2_ = CH_2_(*m*-FC_6_H_4_)) enhances these hydrophobic interactions, as both groups favor nonpolar contacts with the surrounding residues. This compound has a docking score of −6.8 kcal/mol and a longer D_Nitro-N1_ distance of 4.06 Å, compared to compounds **5a** and **1**. 

For the known active ligands, **NFX** exhibits a docking score of −6.5 kcal/mol, with its interactions within the binding site being mediated by hydrophobic interactions with residues A:Y179 and A:L183, and showing a low D_Nitro-N1_ value of 2.6 Å. Compound **2a** establishes a hydrogen bond with residue B:Q147, in addition to hydrophobic interactions with residues A:L183, B:K92, B:Q145, and B:A257, and also presents a low D_Nitro-N1_ value of 2.38 Å. Similarly, compound **2b** exhibits interactions comparable to those of **2a**, with a hydrogen bond observed with residue B:Q147 and hydrophobic interactions with A:L183, B:Q145, and B:A257. It is worth noting that, for both compounds **2a** and **2b**, the hydrogen bond formation involves the same atoms as in compounds **5a** and **1**. Overall, the proximity of the nitro groups to the N1 carbon may be related to the experimentally observed activity. The binding modes for the remaining compounds can be found in Supplementary Figure S13.

#### 2.4.7. General Discussion

It has been reported that optimal activity in nitro compounds with trypanocidal activity is usually associated with intermediate lipophilicity. Excessive lipophilicity could limit bioavailability and hinder cell permeability, while low lipophilicity compromises the compound’s ability to cross the parasite’s cell membrane [44].

**Figure 11:**
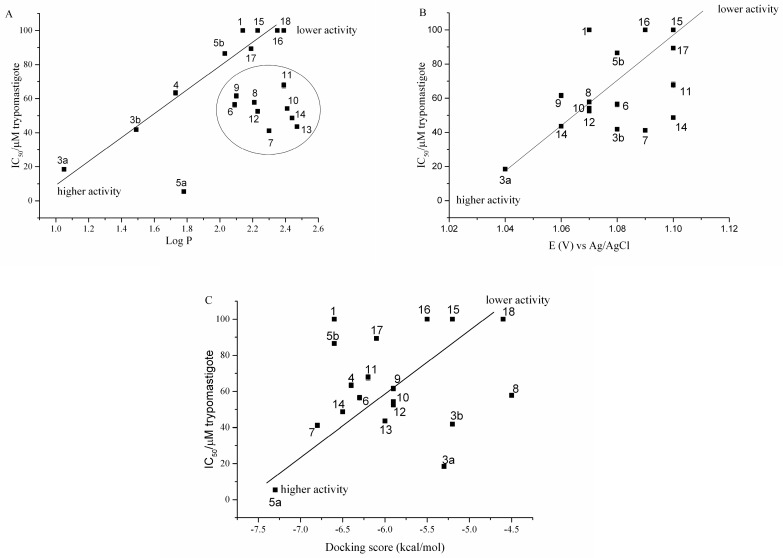
Correlations between (**A**) lipophilicity and trypanocidal activity on the trypomastigote form of the 5-nitroindazolin-3-one series, the halogenated compounds that did not correlate are indicated in a circle, (**B**) reduction potentials (Epc) and trypanocidal activity on the trypomastigote form of the 5-nitroindazolin-3-one series, and (**C**) interaction energy with *Tc*NTR and trypanocidal activity on the trypomastigote form of the 5-nitroindazolin-3-one series.

In the series of 5-nitroindazolin-3-ones, lipophilicity is observed to influence the activity of the compounds significantly. Figure 11A shows compounds with a lower LogP value show higher trypanocidal activity, while those with higher lipophilicity show reduced activity. However, this trend needs to be clarified in compounds containing halogens in their structure (circled in Figure 11A), where the influence of lipophilicity does not directly correlate with trypanocidal activity. The most active compound, **5a**, exhibits intermediate lipophilicity, suggesting that, while lipophilicity is an important parameter, it is not the only determining factor for the design of compounds with high trypanocidal activity.

To correlate trypanocidal activity on the trypomastigote form with the reduction potentials of the 5-nitroindazolin-3-one series (Figure 11B), a low correlation between both parameters was found. For example, compound 3a exhibits high trypanocidal activity, consistent with its ability to generate reactive oxygen species (ROS) by reducing the nitro group. However, the most active compound, 5a, showed a less direct relationship between its reduction potential and activity, despite its high capacity to induce oxidative stress in the parasite. This suggests that the activation of 5a within the parasite does not depend exclusively on its reduction potential but that there may be alternative mechanisms or specific interactions with key enzymes, such as *Tc*NTR, that facilitate the reduction of the nitro group and the subsequent generation of ROS at levels sufficient to induce apoptosis in the parasite.

An attempt was also made to correlate trypanocidal activity with the interaction energies with *Tc*NTR (Figure 11C). Although no direct relationship was found between both parameters, compound **5a** had the highest affinity with the enzyme’s active site, which correlates with its high trypanocidal activity within the series studied. The hydrophilic nature of the active site of *Tc*NTR, responsible for interacting with the highly polar reduced flavin mononucleotide (FMNH_2_), suggests that compounds with lower lipophilicity and greater capacity to form polar interactions may have a better affinity for the active site [11].

In the most active compounds, such as **5a**, it has been observed that the capacity to form interactions with polar residues and the favorable disposition for hydrogen bonds improve their trypanocidal activity. Docking analysis reinforces this idea, suggesting that compounds with substituents that increase polarizability without increasing lipophilicity too much tend to be more effective.

Finally, the discrepancy between the reduction potential of compound **5a** and its ability to generate oxidative stress could be explained by a combination of factors. Although its reduction potential is more negative, its high affinity for *Tc*NTR (evidenced in docking studies) allows it to be efficiently reduced in the enzymatic environment, effectively generating ROS. Furthermore, the moderate lipophilicity of compound **5a** would ensure good bioavailability and cell permeability, contributing to its success as a trypanocidal compound. In summary, the high activity of compound **5a** is not based solely on its electrochemical properties, but on a synergy between its structure, affinity for *Tc*NTR, and activation capacity in the parasite.

## 3. Materials and Methods

### 3.1. Chemistry

#### 3.1.1. General Methods 

Reagent-grade chemicals were purchased from Sigma-Aldrich Chemical Co. (St. Louis, MO, USA) and solvents were purchased from Merck (Darmstadt, Germany). Melting points (mps) were determined using a Stuart Scientific melting point apparatus (SMP3, Cole-Parmer Ltd, Staffordshire, UK). The ^1^H (500 MHz) and ^13^C (125 MHz) NMR spectra of **17**, owing to their complexity (C-F coupling), were recorded at room temperature (≈20 °C) on a Varian System-500 spectrometer (Varian, Inc., Palo Alto, CA, USA). The ^1^H (300 MHz) and ^13^C (75 MHz) NMR spectra of the remaining compounds were recorded at the same temperature on a Bruker Avance-300 spectrometer (Bruker Co., Billerica, MA, USA). The chemical shifts are reported in ppm from TMS (*δ* scale) but were measured against the solvent signal. The assignments were performed by means of different standard homonuclear and heteronuclear correlation experiments (NOE, gHSQC and gHMBC). DC-Alufolien silica gel 60 PF_254_ (Merck, 0.2 mm layer thickness) was used for TLC and silica gel 60 (Merck, particle size 0.040–0.063 mm) for flash column chromatography. Microanalyses were performed on a Heraeus CHN-O-RAPID analyzer (Heraeus, Hanau, Germany); analyses were within ±0.3% of the theoretical values. 

The epimastigotes and trypomastigotes of the Dm28c strain of *T. cruzi* and RAW 264.7 cells were obtained from an in-house collection (Programa de Farmacología Molecular y Clínica, Facultad de Medicina, Universidad de Chile).

The ESR spectra were recorded in the X band (9.7 GHz) using an ECS 106 spectrometer (Bruker, Coventry, UK) with a rectangular cavity and 50 kHz field modulation, equipped with a high-sensitivity resonator at room temperature. Spectrometer conditions were as follows: microwave frequency 9.81 GHz, microwave power 20 mW, modulation amplitude 0.91 G, receiver gain 59 db, time constant 81.92 ms, and conversion time 40.96 ms. The fluorescence was measured with a Varioskan™ LUX multidetection microplate reader from ThermoFisher Scientific (Waltham, MA, USA), using white polystyrene 96-well plates, purchased from Nunc (Roskilde, Denmark). Fluorescence was read from the top, and the plate reader was controlled by SkanIt RE 7.0.2 software.

#### 3.1.2. Preparation of 1,2-Disubstituted Indazolinones

**1**, **5a,b**, and **6** were prepared following the described procedure in [19], closely related to method A. Separately, **3a**, **3b**, and **4** were prepared as described by [15,45,46], respectively.

Method A (applied to the preparation of compounds **7**–**18**): A stirred mixture of the corresponding 2-substituted indazolin-3-one **24** (3.00 mmol), methyl iodide (1.0 mL, excess), and potassium carbonate (0.55 g, 4.00 mmol) in DMF (20 mL) was stirred at RT overnight. The solvent was then evaporated to dryness and, after the addition of chloroform (50 mL), inorganic salts were separated by filtration. The concentrated chloroform phase was applied to a flash silica gel chromatography column which was eluted with chloroform/acetone mixtures (30:1 to 20:1) to afford the desired compounds (Scheme 1).

Method B (applied to the preparation of indazolinones **8**–**12**, **14**, and **17**): A stirred solution of 1-methyl-5-nitroindazol-3-ol **19** (0.96 g, 4.97 mmol) and the corresponding benzyl bromide (11.00 mmol) in DMF (3 mL) was heated for 4 h at 150 °C. The solvent was then evaporated to dryness and, after the addition of water (50 mL), the precipitated solid was collected by filtration. The crude products were applied to a flash silica gel chromatography column which was eluted with chloroform/acetone mixtures (30:1 to 20:1) to afford the main reaction products, the desired 1,2-disubstituted indazolinones (Scheme 1).

#### 3.1.3. Preparation of 5-Nitroindazole 

Compounds **19**, **20**, and **21** were prepared as described [46], while **22** and **23** were prepared as described by [15,47], respectively.

### 3.2. Cyclic Voltammetry (CV)

Cyclic Voltammetry (CV) was carried out using a Metrohm Autolab instrument model PGSTAT204, using dimethyl sulfoxide (DMSO) as the solvent (1 mM solutions), tetrabutylammonium perchlorate (0.1 M TBAP) as the supporting electrolyte, a hanging drop mercury electrode (HDME) as the working electrode, Ag/AgCl (3M KCl) as the reference electrode, and a graphite rod as the counter electrode. All measurements were performed after bubbling with nitrogen (N_2_) for 10 min.

Using Nicholson’s procedure [28], the Ipa/Ipc values (RNO_2_/RNO_2_^•−^) were measured from each cyclic voltammogram with a scan rate between 0.1 and 2.5 V/s. 

### 3.3. ESR Spectroscopy

ESR spectra were recorded in the X band (9.85 GHz) using a Bruker ECS106 spectrometer with a rectangular cavity and 50 kHz field modulation. The hyperfine splitting constants were estimated to be accurate within 0.05 G. The anion radicals were generated by electrolytic reduction in situ using DMSO as a solvent and TBAP as a supporting electrolyte. All experiments were conducted at room temperature, under a nitrogen atmosphere, and at the same reduction potential found in the CV experiments. The ESR spectra were simulated using the program EPR-WinSIM Version 0.98.

### 3.4. Biologic Assays

#### 3.4.1. Cytotoxicity Assay 

The effect of compound treatments on RAW 264.7 cells (ATCC TIB-71^®^) was evaluated through the tetrazolium dye 3-(4,5-dimethylthiazol-2-yl)-2,5-diphenyltetrazolium bromide (MTT) assay as a viability test [48]. The compounds under study, dissolved in dimethylsulfoxide (DMSO), were added to the culture media at 10–200 μM.

RAW 264.7 cells were cultured in 96-well plates (5 × 10^4^ cells per well), and 100 μL of 0.5 mg/mL MTT was added to each well. The DMSO final concentration was less than 0.1% *v*/*v*. After incubation for 4 h at 37 °C, the generated water-insoluble formazan dye was dissolved by adding 100 μL of 10% *w*/*v* sodium dodecyl sulfate (SDS) in 0.01 M HCl. The plates were further incubated overnight at 37 °C; the generated water-insoluble formazan dye was dissolved by adding 100 μL of 10% *w*/*v* sodium dodecyl sulfate (SDS) in 0.01 M HCl, and 0.5% of Triton X-100 was added as a positive control. The plates were further incubated overnight at 37 °C, and the optical density of the wells was determined using a microplate reader (Asys Expert Plus©, Asys Hitachi, Vienna, Austria) at 570 nm. Under these conditions, the optical density is directly proportional to the viable cell number in each well. All experiments were performed at least thrice and data were reported as means and their standard deviations from triplicate cultures. Results are reported as IC_50_ regarding the control (uninfected cells in culture medium)

#### 3.4.2. Epimastigotes Viability Study 

The trypanocidal activity was evaluated against the *T. cruzi* epimastigote stage (Dm28c strain). It was measured through the MTT assay using 0.22 mg/mL phenazine methosulfate (as an electron carrier). *T. cruzi* epimastigote from the author’s collection (Programa de Farmacología Molecular y Clínica, Facultad de Medicina, Universidad de Chile, Santiago, Chile) were grown at 28 °C in liver infusion tryptase (LIT) medium, as reported earlier [49], but replacing blood with 4 μM hemin. Inactived fetal bovine serum (FBS) was added to a final concentration of 10% *v*/*v*. In this colorimetric assay for testing the trypanocidal activity, the compounds were dissolved in DMSO. They were added to 3 × 10^6^ parasites/mL at 100 μM final concentrations in RPMI 1640 culture medium without phenol red for 24 h at 28 °C.

The final concentration of DMSO was less than 0.1% *v*/*v*. Likewise, NFX and BNZ (1 mM) were added as positive controls. The MTT assay, which was described previously, determined the viability of the epimastigotes.

#### 3.4.3. Trypomastigote Viability Study 

Vero cells (ATCC CCL-81) were infected with Dm28c trypomastigotes at a 1:3 (cell: parasite) ratio. *T. cruzi* trypomastigotes were initially obtained from primary cultures of peritoneal macrophage from chagasic mice. Vero cells were cultured in 5% inactive fetal bovine serum supplemented RPMI 1640 medium in humidified air with 5% CO_2_ at 37 °C. Vero cell cultures were then infected with trypomastigotes and incubated at 37 °C in humidified air and 5% CO_2_ for 5–7 days. After that time, the culture medium was collected and centrifuged at 500× *g* for 5 min. The trypomastigote-containing pellet was re-suspended in RPMI 1640 supplemented with inactivated FBS and penicillin–streptomycin at a final 1 × 10^7^ parasites/mL density. Trypomastigote viability assays were performed using the MTT reduction method as described previously. Then, 1 × 10^7^ parasites/mL was incubated in RPMI 1640 with inactivated FBS at 37 °C for 24 h with or without the studied compounds. 

#### 3.4.4. Plasmatic Membrane Permeability Assay

Late growth-phase trypomastigotes of *T. cruzi* (3 × 10^6^/well) were washed and incubated in the dark with 1 μM SYTOX Green probe (Molecular Probes) in HANKS′ balanced salts solution (HBSS; Sigma-Aldrich) supplemented with 10 mM D-glucose (HBSS + Glu). The test compound was added at IC_50_, and fluorescence was measured after 1 h of treatment [50]. The maximum permeabilization was obtained with 0.5% Triton X-100. Fluorescence intensity was determined using Varioskan™ LUX multimode microplate reader (ThermoFisher Scientific, Waltham, MA, USA), with excitation and emission wavelengths of 485 and 520 nm, respectively. The following internal controls were used in the evaluation: (i) the background fluorescence of the compound at the respective wavelengths and (ii) the possible interference of DMSO. Samples were tested in triplicate [51].

#### 3.4.5. Analysis of ATP Levels

The CellTiter-Glo^®^ Luminescent Cell Viability Assay (Promega, Fitchburg, WI, USA) was used to detect variations in ATP levels. After incubation, treated parasites were centrifuged (6000 rpm, 10 min, 4 °C) and treated according to the manufacturer’s instructions. Luminescence was determined using the Varioskan™ LUX multi-mode microplate reader (ThermoFisher Scientific). Results were expressed as a percentage relative to the negative control without treatment. Sodium azide (20 mM NaN_3_ for 3 h) was added as a positive control and benznidazole as a reference treatment [36].

(1) $$\%relative\;control=\frac{luminescence\;sample}{luminescence\;control}*100$$

#### 3.4.6. Intracellular Generation of Reactive Oxygen Species (ROS)

ROS detection was performed using the 2′, 7′-dichlorodihydrofluorescein diacetate (DCFH_2_-DA) method. For Dm28c trypomastigotes, 96-well plates were seeded with 10 × 10^6^ trypomastigotes/mL in RPMI medium without phenol red and supplemented with 5% SFBi. Cultures were incubated with a 20 μM DCFH_2_-DA solution for 15 min at 37 °C. Then, they were centrifuged at 6000 rpm and washed 2 times with phosphate-buffered saline at pH 7.4. The DCFH_2_-DA-loaded trypomastigotes were then transferred to a Nunc^®^ fluorescence 96-well plate.

On the other hand, 5 × 10^4^ VERO cells were seeded in Nunc^®^ fluorescence 96-well plates in RPMI medium without phenol red, supplemented with 5% inactivated SFB, and incubated for 24 h at 37 °C and 5% CO_2_. Then, the culture medium was replaced, and the 20 μM DCFH_2_-DA solution was added and incubated for 15 min at 37 °C. After this time, the cells were washed with PBS pH 7.4.

Subsequently, the most active compounds were added at the IC_50_ concentration in trypomastigotes and fluorescence (excitation: 488 nm, emission: 528 nm) was recorded for 40 min in a Biotek Synergy HT spectrofluorometer. The area under fluorescence increase over time curves were determined using Origin 8 software 9.2, with areas normalized to control. Results are the means ± SD of three independent experiments.

#### 3.4.7. Determination of the Effect on Mitochondrial Membrane Potential (Δψm) in Dm28c Trypomastigote of *T. cruzi*

Mitochondrial membrane potential (ψm) was determined by the incorporation of the fluorescent probe tetramethylrhodamine methyl ester (TMRM) [52]. Trypomastigotes of the Dm28c strain (1 × 10^7^ parasites/mL) were seeded in a 96-well plate, with the most active compounds added to the concentration of IC_50_. Then, carbonylcyanide-m-chlorophenylhydrazone (CCCP, 10 µM) was added as a control and incubated at 37 °C in humidified air and 5% CO_2_ for 3 h. Parasites were washed with phosphate-buffered saline (pH 7.4), and suspensions were incubated with TMRM (50 nM) for 15 min. Fluorescence measurements determined the incorporation of the probe at an excitation wavelength of 550 nm and an emission of 590 nm. All the assays were performed in darkness.

#### 3.4.8. ESR Studies in Parasite Media

The free radical production capacity of the new complexes was assessed in the parasite by ESR using 5,5- dimethyl-1-pirroline-N-oxide (DMPO) for spin trapping [53]. Each tested compound was dissolved in DMSO (spectroscopy grade) (ca. 1 mM) and the solution was added to a mixture containing the trypomastigote form of *T. cruzi* (Dm28c strain, final protein concentration 4–8 mg/mL) and DMPO (final concentration 250 mM). The mixture was transferred to a 50 μL capillary. ESR spectra were recorded in the X band (9.85 GHz) using a Bruker ECS 106 spectrometer with a rectangular cavity and 50 KHz field modulation. All the spectra were registered in the same scale after 15 scans.

#### 3.4.9. Interaction of Nitro Compounds with TcNTR through Docking Calculations

We performed molecular docking calculations to understand the potential interaction of the compounds **1** to **23** and NFX with the *T. cruzi* nitroreductase protein including reduced flavin mononucleotide (FMNH_2_) as cofactor in the binding site. Structure modeling of the protein was developed using the ESMFold v1.0 algorithm [54] and the sequence was retrieved from the UniProt [55] database (I6Q1A4 code). The residues 1–14 were removed because they are predicted to carry a signal peptide [10]. Compounds were prepared using Openbabel software v3.1.0 [56]. The Autodock-Vina 1.2.0 package [57] was employed for the docking calculations, setting a box size of 35 × 35 × 35 Å^3^ of size around the binding site with a grid space of 0.375 Å. The binding site was defined next to the flavin mononucleotide (FMN) compound reported in the crystallographic structure of the *E. coli* Nitroreductase receptor (PDB ID:1DS7, located in the dimeric interface). Additionally, 1000 runs of the Lamarckian Genetic Algorithm and a population size of 1000 were employed in the protocol. The non-bonding interactions of the docked protein–compound complexes were estimated using the Protein–Ligand Interaction Profiler (PLIP) server [58]. 

### 3.5. Statistical Analysis

Statistical analysis was performed using Graph Pad Prism 9.5.0 (GraphPad Software. San Diego, CA, USA). Data are expressed as mean ± SD of three independent experiments. Determination of IC_50_ values was performed by sigmoidal curve interpolation on a 6-point curve, with an R-squared greater than 0.9500.

Statistical analysis was performed using one-way ANOVA with Dunnett post-test when multiple comparisons were required. Data are considered statistically significant when *p* < 0.05. 

## 4. Conclusions

In this study, a series of 5-nitroindazolin-3one derivatives were synthesized and evaluated against *Trypanosoma cruzi*, establishing a relationship between the chemical structure and the biological activity of the compounds. The results indicated that the presence of the nitro group is essential for trypanocidal activity; however, the incorporation of electroattracting groups, such as trifluoromethyl in compound **17**, significantly reduced the effectiveness against trypomastigotes.

Based on the analysis of the structure–activity relationship, it is promising to investigate the introduction of substituents that increase the compound’s lipophilicity without affecting its ability to be reduced by the enzyme *Tc*NTR, a key factor in the activation of nitro compounds. In particular, it would be interesting to replace the trifluoromethyl group with substituents that offer an optimized balance between lipophilicity and electron-withdrawing capacity, such as smaller alkyl groups or heterocycles, which could improve both bioavailability and affinity for the parasite’s enzymes. Additionally, it would be valuable to explore the influence of the position of the nitro group in the indazolin-3-one skeleton, as well as its effect on reduction potentials and interaction with *Tc*NTR.

## Figures and Tables

**Table 1:** Voltametric parameters corresponding to reduction of nitro group of derived 5-nitroindazoles, for IIIc/IIIa vs. Ag/AgCl electrode in DMSO.

Compound	−E_pcIII_ (V)	−E_paIII_ (V)	−E_1/2_ (V)	Ipa/Ipc	Compound	−E_pcIII_ (V)	−E_paIII_ (V)	−E_1/2_ (V)	Ipa/Ipc
**1**	1.12	1.02	1.07	0.94	**13**	1.13	0.99	1.06	0.97
**3a**	1.13	0.95	1.04	0.99	**14**	1.20	0.99	1.10	0.94
**3b**	1.13	1.03	1.08	1.00	**15**	1.16	1.04	1.10	0.96
**4**	1.37	1.27	1.32	0.63	**16**	1.15	1.03	1.09	0.91
**5a**	1.36	1.32	1.34	0.62	**17**	1.37	0.82	1.10	0.96
**5b**	1.12	1.04	1.08	0.97	**18**	1.12	1.02	1.07	0.91
**6**	1.22	0.94	1.08	0.99	**19**	1.35	1.28	1.32	0.65
**7**	1.20	0.98	1.09	1.00	**20**	1.38	1.28	1.33	0.67
**8**	1.14	0.99	1.07	1.00	**21**	1.37	1.27	1.32	0.56
**9**	1.10	1.02	1.06	0.96	**22**	1.38	1.26	1.33	0.59
**10**	1.17	0.96	1.07	0.93	**23**	1.37	1.25	1.31	0.78
**11**	1.21	0.99	1.10	0.89	**NFX**	0.91	0.85	0.88	1.01
**12**	1.17	0.96	1.07	1.00	**BNZ **	1.08	0.93	1.00	0.98

**Table 2:** Hyperfine coupling constants of 5-nitroindazoles and 5-nitroindazolone obtained by semi-empirical simulation.

Compounds	aN_1_	aN_2_	aH_1_	aH_2_	aH_3_
**1**	10.28	1.42	4.57	2.42	0.97
**3a**	10.42	1.39	4.60	2.39	1.05
**3b**	11.01	1.26	5.30	2.31	1.01
**4**	11.04	1.28	5.30	2.29	0.95
**5a**	11.67	1.20	5.25	2.37	1.01
**5b**	11.51	1.21	5.01	2.31	1.06
**6**	10.95	1.23	4.97	2.28	1.05
**7**	11.09	1.20	5.31	2.27	0.98
**8**	11.15	1.17	5.19	2.41	0.95
**9**	11.27	1.28	5.21	2.38	0.86
**10**	10.11	1.25	5.18	2.43	1.96
**11**	11.04	1.27	5.24	2.28	1.05
**12**	11.17	1.21	5.19	2.40	1.11
**13**	11.32	1.24	5.20	2.37	1.04
**14**	11.41	1.18	5.17	2.29	0.94
**15**	11.36	1.21	5.15	2.37	1.01
**16**	11.06	1.23	5.20	2.41	1.04
**17**	11.12	1.18	5.26	2.39	1.01
**18**	11.09	1.21	5.21	2.41	1.10
**19**	12.29		4.55	2.03	2.03
**20**	12.43		4.69	2.08	2.08
**21**	12.36		4.77	1.77	1.77
**22**	12.31		4.62	2.09	2.09
**23**	12.33		4.70	2.05	2.05

**Table 3:** In vitro cytotoxicity in murine RAW 264.7 macrophages (IC_50_) and trypanocidal activity in epimastigote and trypomastigote forms. The IC_50_ curves obtained are shown in the Supplementary Material Figures S14–S16.

	IC_50_ (µM)					
	RAW 264.7	Epimastigote	Trypomastigote	SI * Epimastigote	SI * Trypomastigote	iLogP **
**1**	37.8 ± 1.2	10.1 ± 0.6	>100	3.7	-	2.14
**3a**	26.1 ± 0.8	8.9 ± 1.3	18.5 ± 0.9	2.9	1.4	1.05
**3b**	>200	28.6 ± 2.1	41.8 ± 1.1	>22.5	>4.8	1.49
**4**	97.5 ± 1.2	46.9 ± 3.4	63.4 ± 1.4	2.1	1.5	1.73
**5a**	68.9 ± 0.7	1.1 ± 0.3	5.4 ± 0.7	62.6	12.8	1.78
**5b**	76.5 ± 1.2	52.2 ± 1.3	86.5 ± 1.2	1.5	0.9	2.03
**6**	83.9 ± 0.9	32.9 ± 0.7	56.4 ± 1.5	2.6	1.5	2.09
**7**	95.1 ± 1.1	29.5 ± 1.5	41.2 ± 0.8	3.2	2.3	2.30
**8**	116.4 ± 1.7	36.4 ± 0.8	57.8 ± 1.3	3.2	2.0	2.21
**9**	175.5 ± 2.3	43.7 ± 2.0	61.6 ± 1.4	4.0	2.8	2.10
**10**	>200	40.3 ± 2.0	54.2 ± 1.1	>5.0	>3.7	2.41
**11**	68.8 ± 1.2	70.1 ± 2.1	67.8 ± 1.7	1.0	1.0	2.39
**12**	72.4 ± 1.6	30.3 ± 1.1	52.5 ± 1.3	2.4	1.4	2.23
**13**	>200	79.8 ± 2.3	43.6 ± 1.1	>2.5	>4.6	2.47
**14**	172.2 ± 2.3	83.9 ± 1.9	48.6 ± 1.2	2.1	3.5	2.44
**15**	36.3 ± 1.1	>100	>100	-	-	2.23
**16**	>200	>100	>100	-	-	2.35
**17**	165.7 ± 2.3	35.4 ± 1.1	89.3 ± 1.0	4.7	1.9	2.19
**18**	>200	68.7 ± 1.7	>100	>2.9	-	2.39
**19**	>200	>100	>100	-	-	1.29
**20**	>200	>100	>100	-	-	1.72
**21**	>200	84.3 ± 2.1	91.2 ± 2.2	>2.4	>2.1	2.33
**22**	>200	6.2 ± 0.9	15.3 ± 1.2	>32.3	>13.1	1.93
**23**	>200	>100	>100	-	-	1.38
**NFX**	263.4 ± 2.2	17.4 ± 1.3	21.5 ± 1.4	15.1	12.3	0.71

* Selectivity Index IC_50_ macrophages RAW 264.7/IC_50_ epimastigotes Dm28c or IC_50_ trypomastigotes Dm28c. ** Molinspiration property engine v2022.08.

**Table 4:** Computational docking scores on nitroreductase of *T. cruzi*.

Compounds	Docking Score (kcal/mol)	Compounds	Docking Score (kcal/mol)
**1**	−6.6	**12**	−5.9
**2a**	−7.4	**13**	−6.0
**2b**	−6.9	**14**	−6.5
**3a**	−5.3	**15**	−5.2
**3b**	−5.2	**16**	−5.5
**4**	−6.4	**17**	−6.1
**5a**	−7.3	**18**	−4.6
**5b**	−6.6	**19**	−5.8
**6**	−6.3	**20**	−5.6
**7**	−6.8	**21**	−6.0
**8**	−4.5	**22**	−6.4
**9**	−5.9	**23**	−5.0
**10**	−5.9	**NFX**	−6.5
**11**	−6.2		

## Data Availability

The original contributions presented in the study are included in the article/Supplementary Material, further inquiries can be directed to the corresponding authors.

## Supplementary Materials

The following supporting information can be downloaded at: https://www.mdpi.com/article/10.3390/ijms252011107/s1.

## Abbreviations

Bn: Benzyl: CH_2_: Methylene group, CH_3_: Methyl group, OCH_3_: Methoxy group, NCH_3_: Methyl group attached to nitrogen, d: Doublet, dd: Doublet of doublets, m: Multiplet, J: Coupling constant, Hz: Hertz (unit of frequency), δ: Chemical shift in parts per million (ppm), Mp: Melting point, 2-PrOH: 2-Propanol (Isopropanol), ^1^H NMR: Proton Nuclear Magnetic Resonance, ^13^C NMR: Carbon-13 Nuclear Magnetic Resonance calcd: Calculated, Found: Experimental analysis result (often for elemental analysis).
