# Supplementary material for: In Vitro Evaluation of New 5-Nitroindazolin-3-one Derivatives as Promising Agents against Trypanosoma cruzi
Source: Int J Mol Sci. 2024 Oct 16;25(20):11107. doi: 10.3390/ijms252011107 (PMC11508334; doi:10.3390/ijms252011107)
Supplement: Supplementary file 1 [file ijms-25-11107-s001.zip › ijms-3247828-supplementary.pdf]

Supplementary Information

In Vitro Evaluation of New 5-Nitroindazolin-3-one Derivatives as Promising Agents against Trypanosoma cruzi

Josué Pozo-Martínez [1,2], Vicente J. Arán [3], Matías Zúñiga-Bustos [4], Sebastián Parra-Magna [4,5], Esteban Rocha-Valderrama [4,5], Ana Liempi [6], Christian Castillo [6], Claudio Olea-Azar [1,*] and Mauricio Moncada-Basualto [4,*]

1 Department of Molecular Pharmacology and Clinical, Faculty of Medicine, University of Chile, Santiago 8380453, Chile; josue.pozo@uazuay.edu.ec
2 Laboratorio de Química-Médica, Facultad de Ciencia y Tecnología, Universidad del Azuay, Av. 24 de Mayo 777, Cuenca 010204, Ecuador
3 Instituto de Química Médica (CSIC), Juan de la Cierva 3, 28006 Madrid, Spain; uvejotaran@gmail.com
4 Instituto Universitario de Investigación y Desarrollo Tecnológico, Universidad Tecnológica Metropolitana,
Santiago 8940577, Chile; mzunigab@utem.cl (M.Z.-B.)
5 Free Radical and Antioxidants Laboratory, Inorganic and Analytical Department, Faculty of Chemical and Pharmaceutical Sciences, University of Chile, Santiago 8380492, Chile
6 Programa de Biología Integrativa, Instituto de Ciencias Biomédicas, Facultad de Medicina, Universidad de Chile, Santiago 8380453, Chile; ccastillor@uchile.cl (C.C.)
* Correspondence: colea@uchile.cl (C.O.-A.); mmoncadab@utem.cl (M.M.-B.)

Figure S1- S12: NMR Spectra of compounds

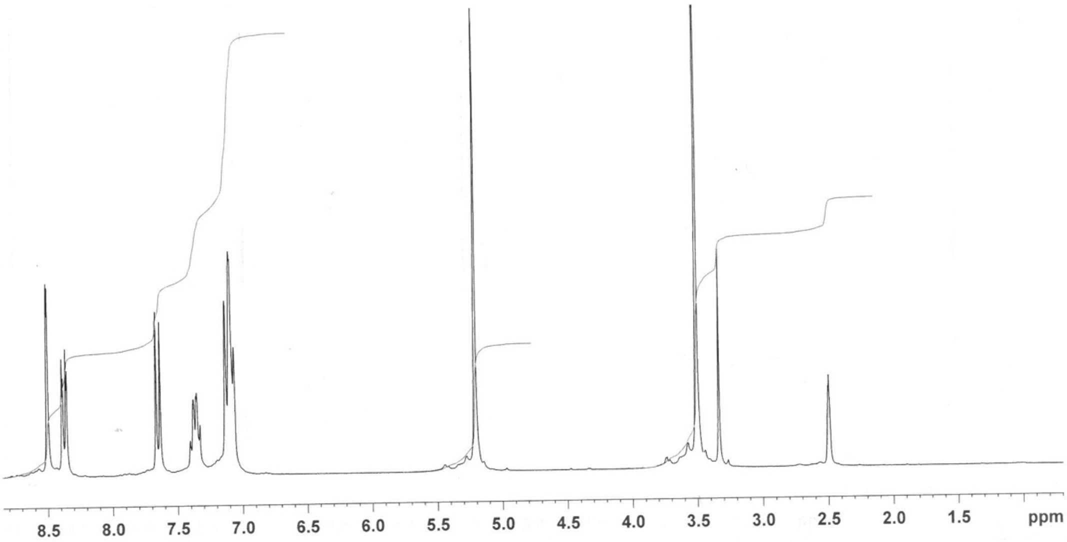
8.5 8.0 7.5 7.0 6.5 6.0 5.5 5.0 4.5 4.0 3.5 3.0 2.5 2.0 1.5 ppm

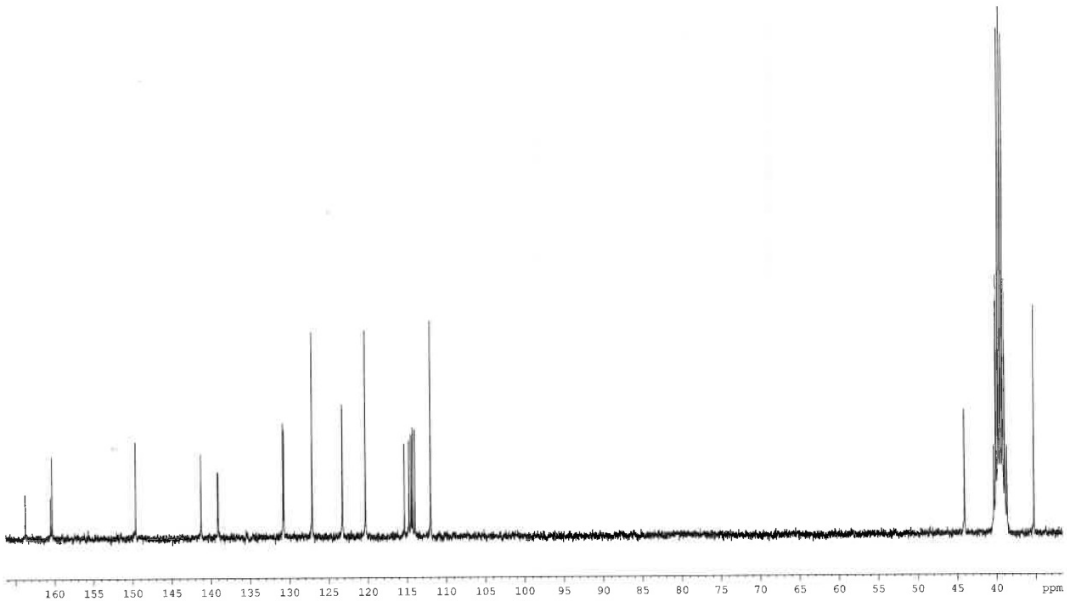

Figure S1. ^{1}H and ^{13}C NMR spectra of compound **7**.

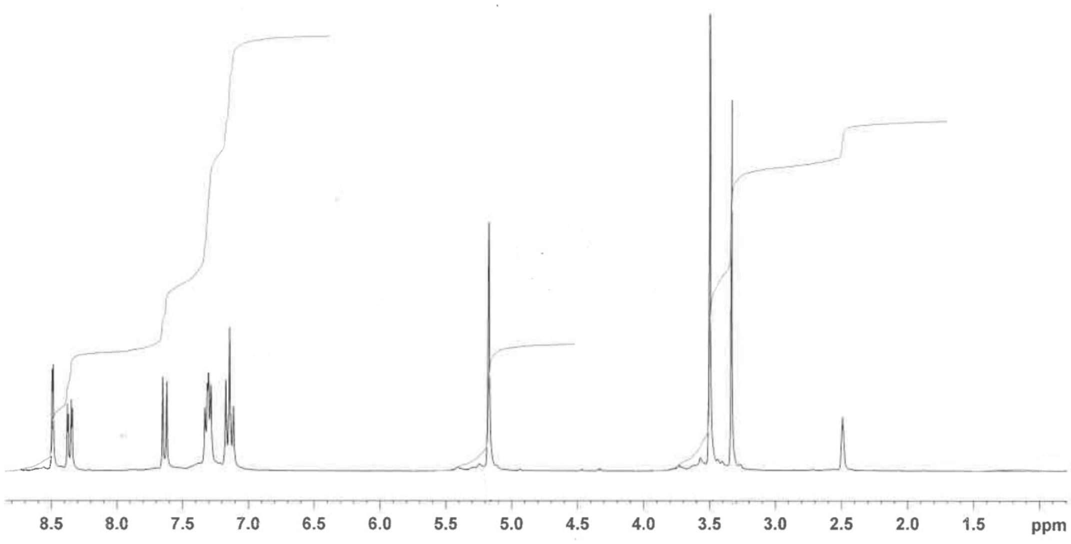
8.5 8.0 7.5 7.0 6.5 6.0 5.5 5.0 4.5 4.0 3.5 3.0 2.5 2.0 1.5 ppm

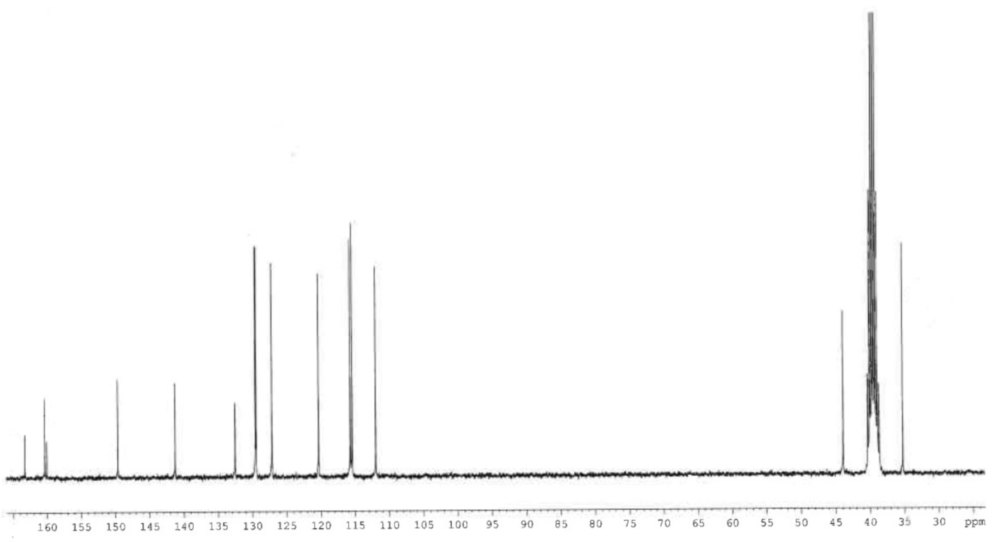

Figure S2. ¹H and ¹³C NMR spectra of compound **8**.

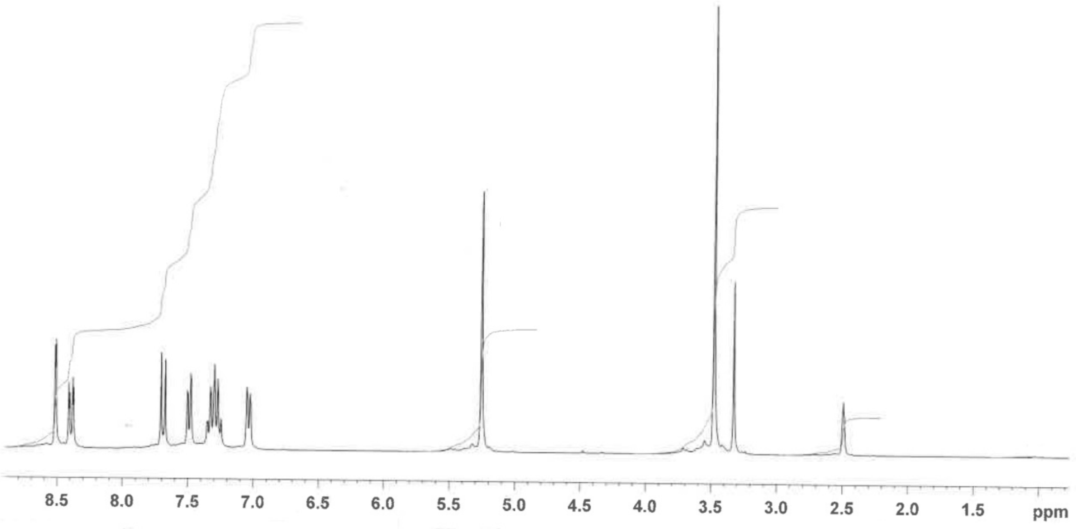
8.5 8.0 7.5 7.0 6.5 6.0 5.5 5.0 4.5 4.0 3.5 3.0 2.5 2.0 1.5 ppm

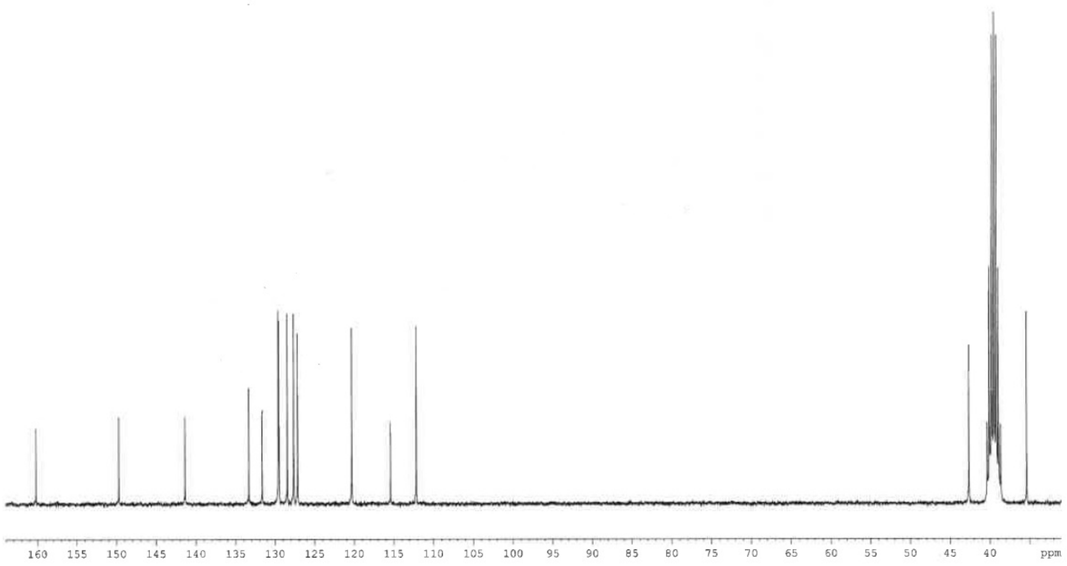

Figure S3. ^{1}H and ^{13}C NMR spectra of compound **9**.

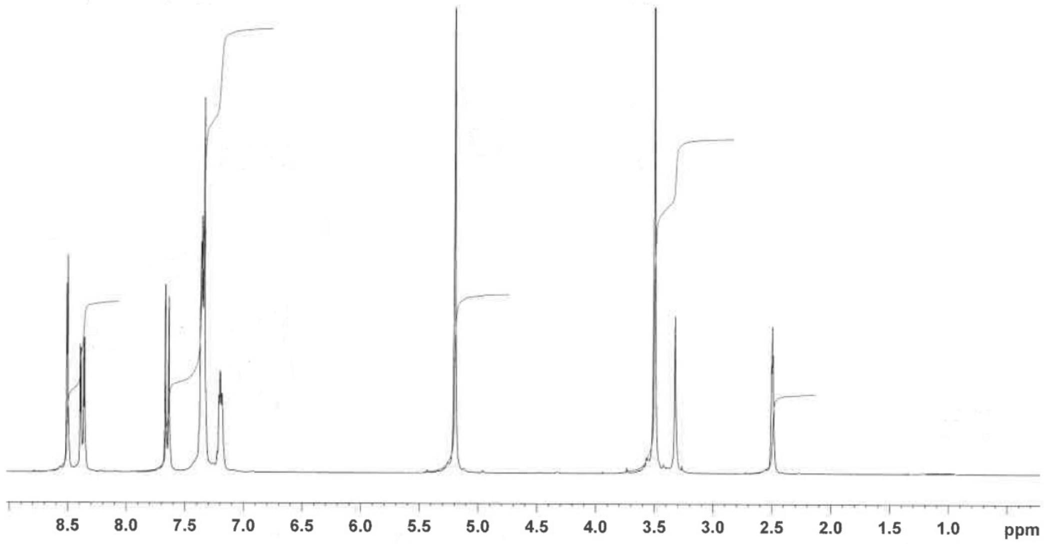
8.5 8.0 7.5 7.0 6.5 6.0 5.5 5.0 4.5 4.0 3.5 3.0 2.5 2.0 1.5 1.0 ppm

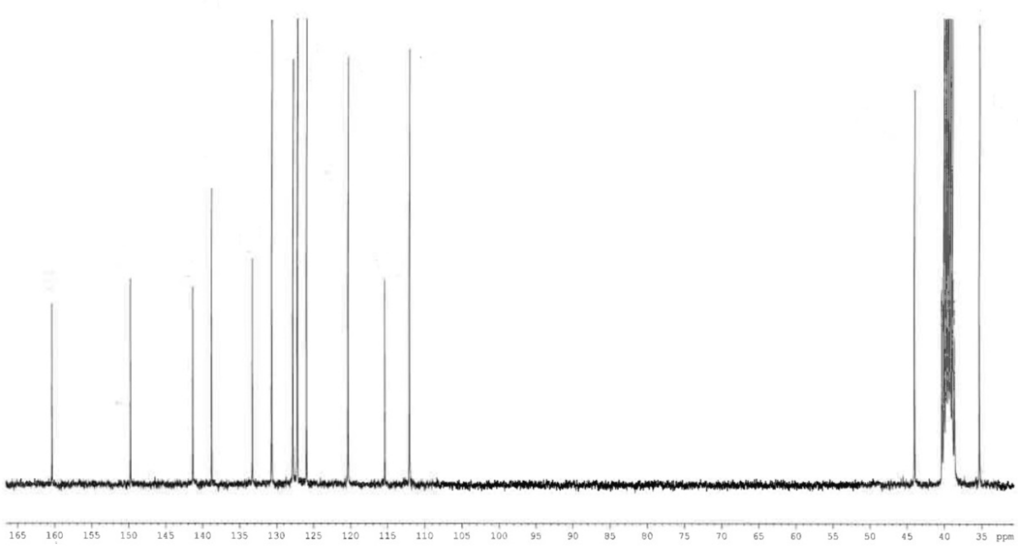

Figure S4. ^{1}H and ^{13}C NMR spectra of compound **10**.

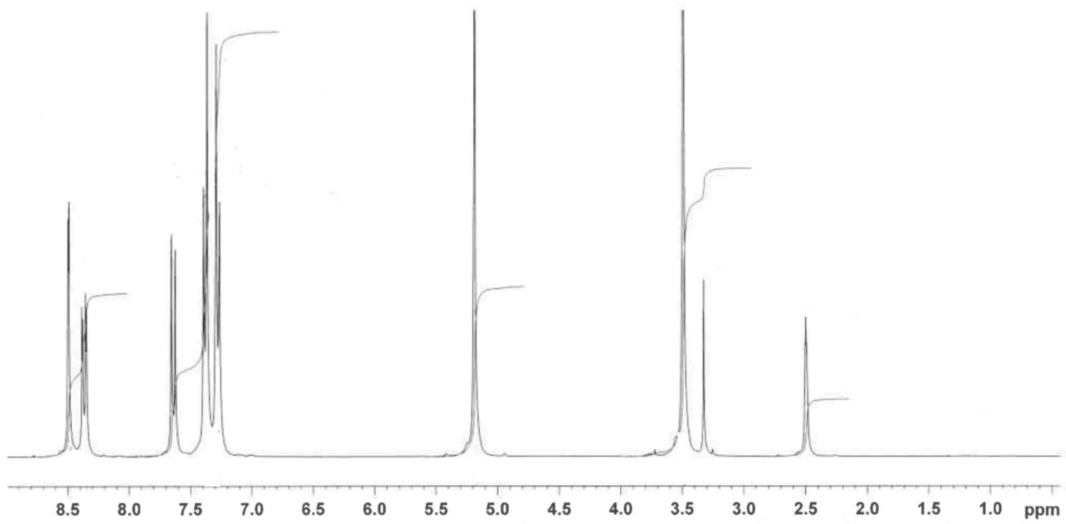
8.5 8.0 7.5 7.0 6.5 6.0 5.5 5.0 4.5 4.0 3.5 3.0 2.5 2.0 1.5 1.0 ppm

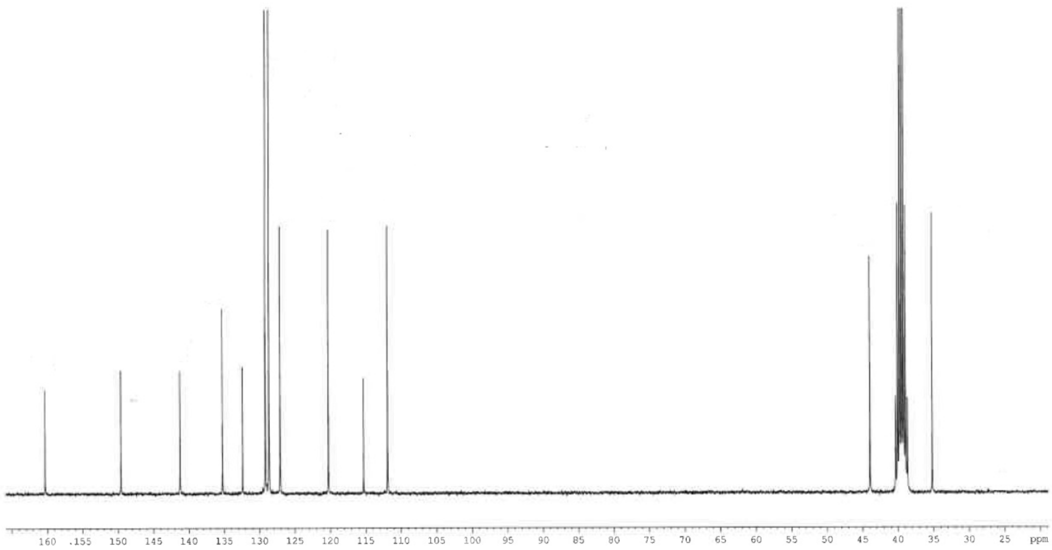

Figure S5. ^{1}H and ^{13}C NMR spectra of compound **11**.

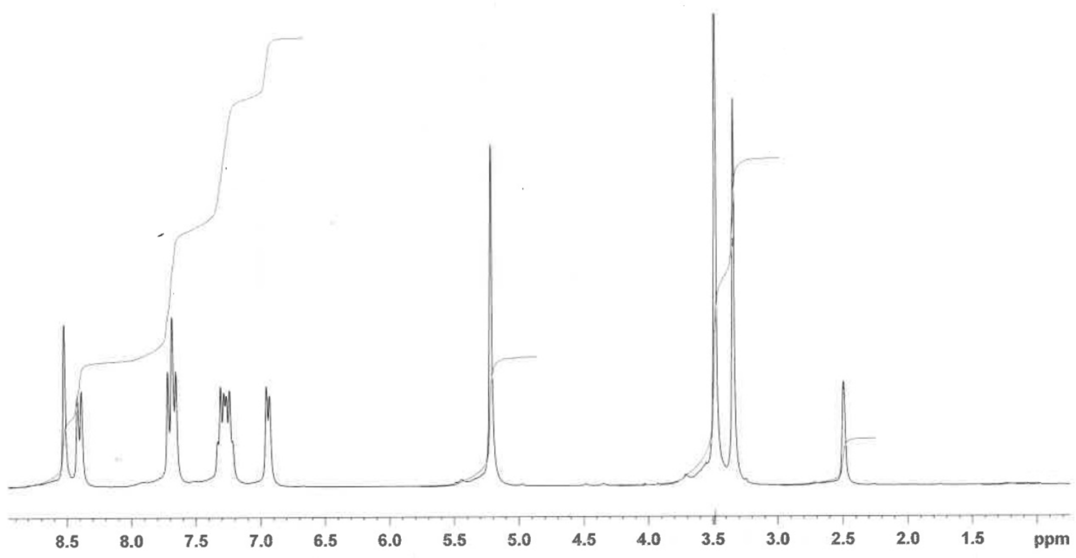
8.5 8.0 7.5 7.0 6.5 6.0 5.5 5.0 4.5 4.0 3.5 3.0 2.5 2.0 1.5 ppm

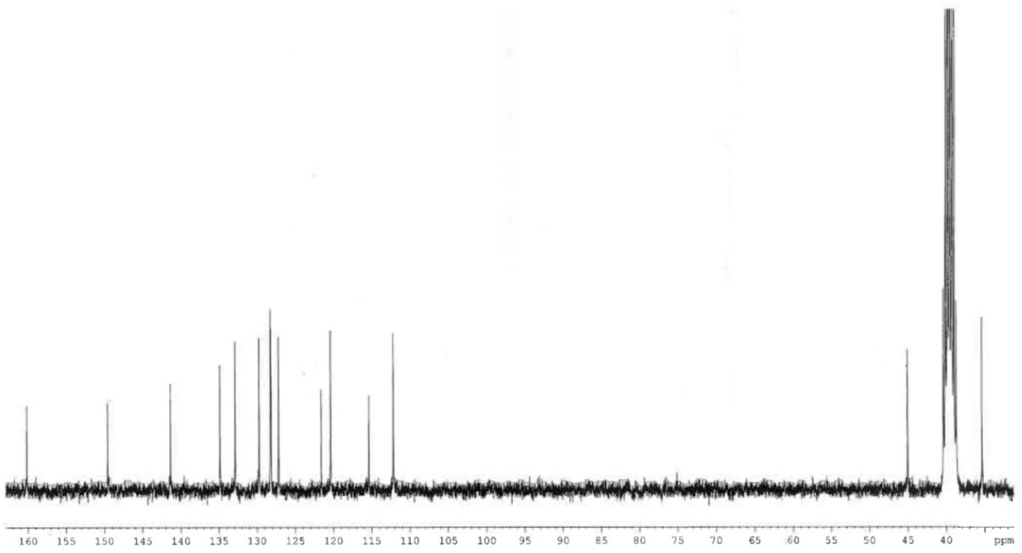
160 155 150 145 140 135 130 125 120 115 110 105 100 95 90 85 80 75 70 65 60 55 50 45 40 ppm

Figure S6. ^{1}H and ^{13}C NMR spectra of compound **12**.

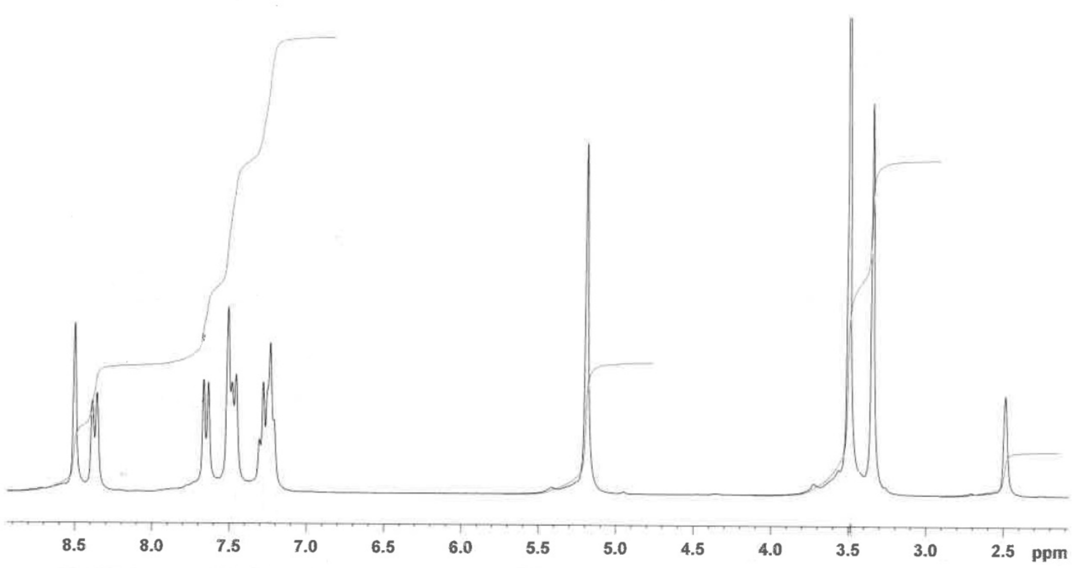

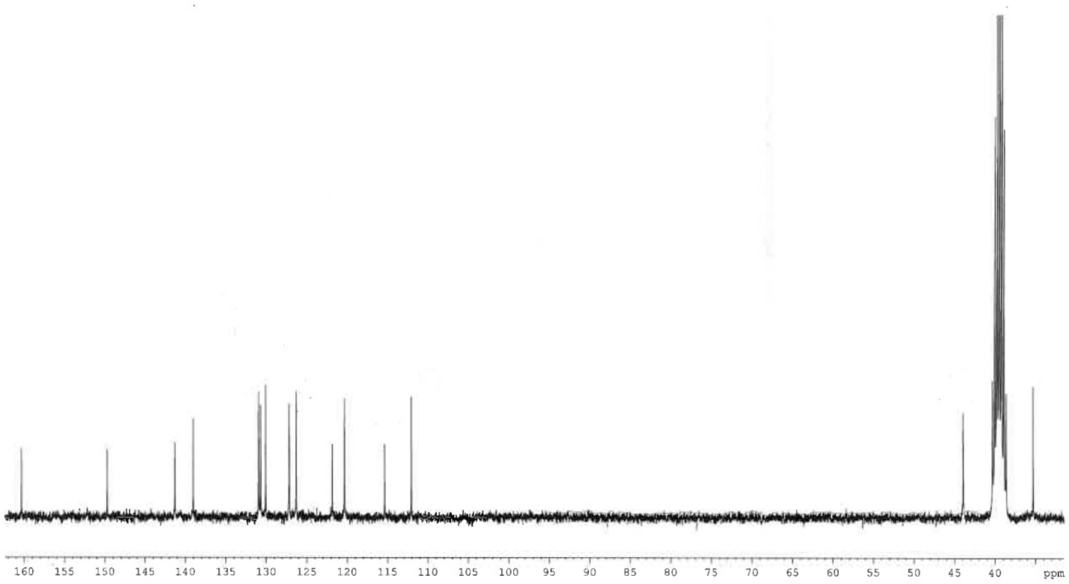

Figure S7. ^{1}H and ^{13}C NMR spectra of compound **13**.

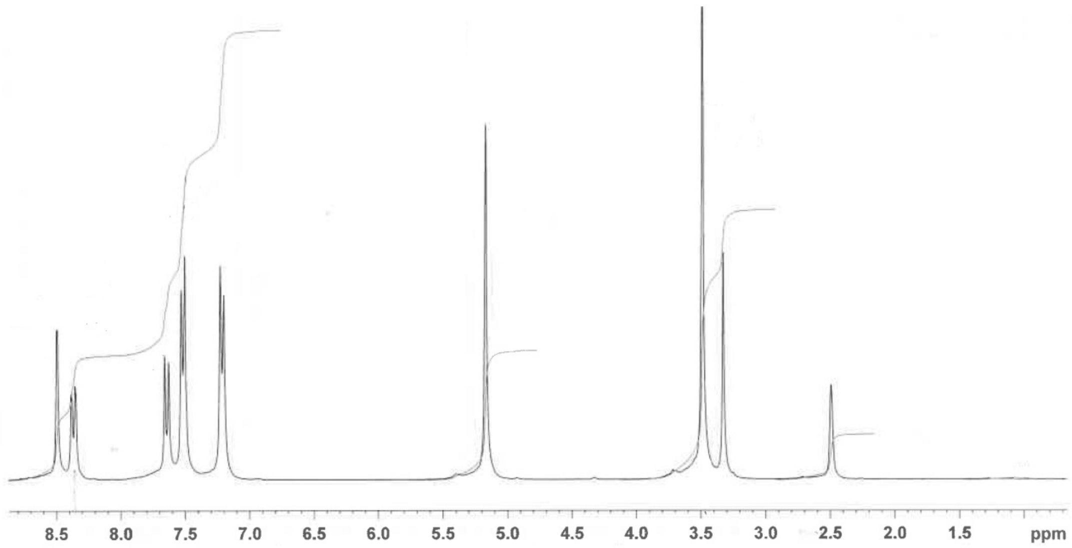

8.5
8.0
7.5
7.0
6.5
6.0
5.5
5.0
4.5
4.0
3.5
3.0
2.5
2.0
1.5
ppm

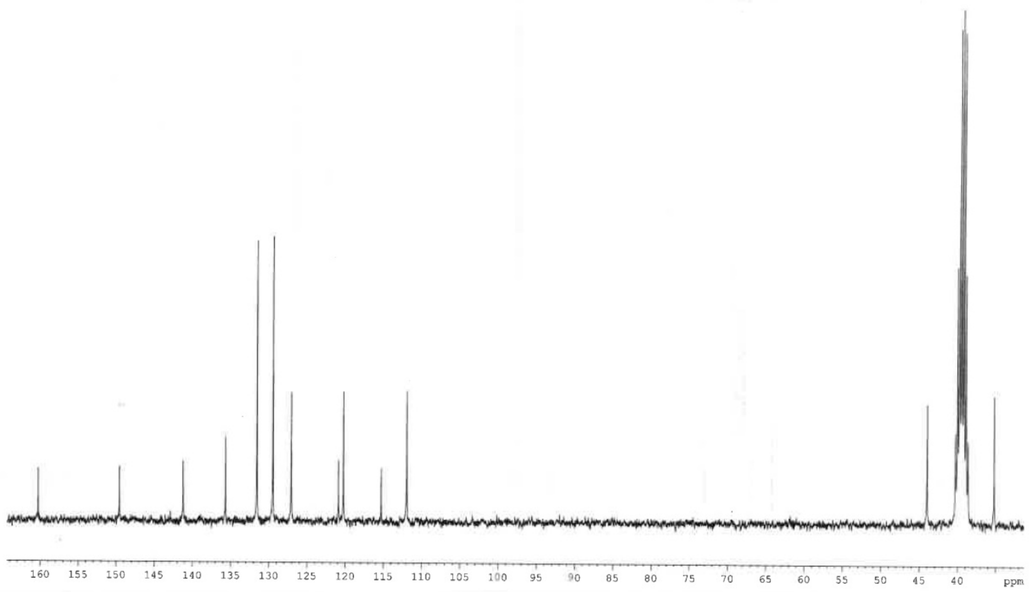

Figure S8. ^{1}H and ^{13}C NMR spectra of compound **14.**

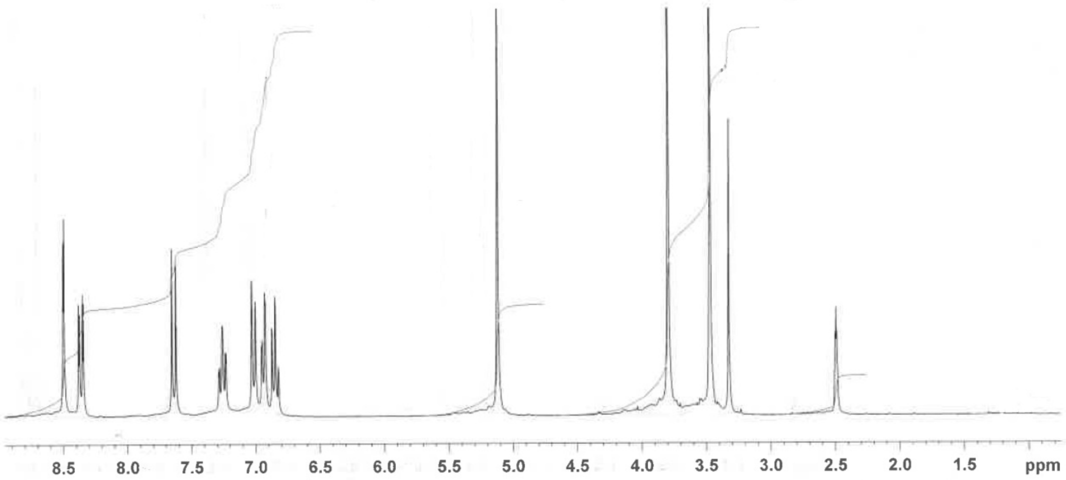
8.5
8.0
7.5
7.0
6.5
6.0
5.5
5.0
4.5
4.0
3.5
3.0
2.5
2.0
1.5
ppm

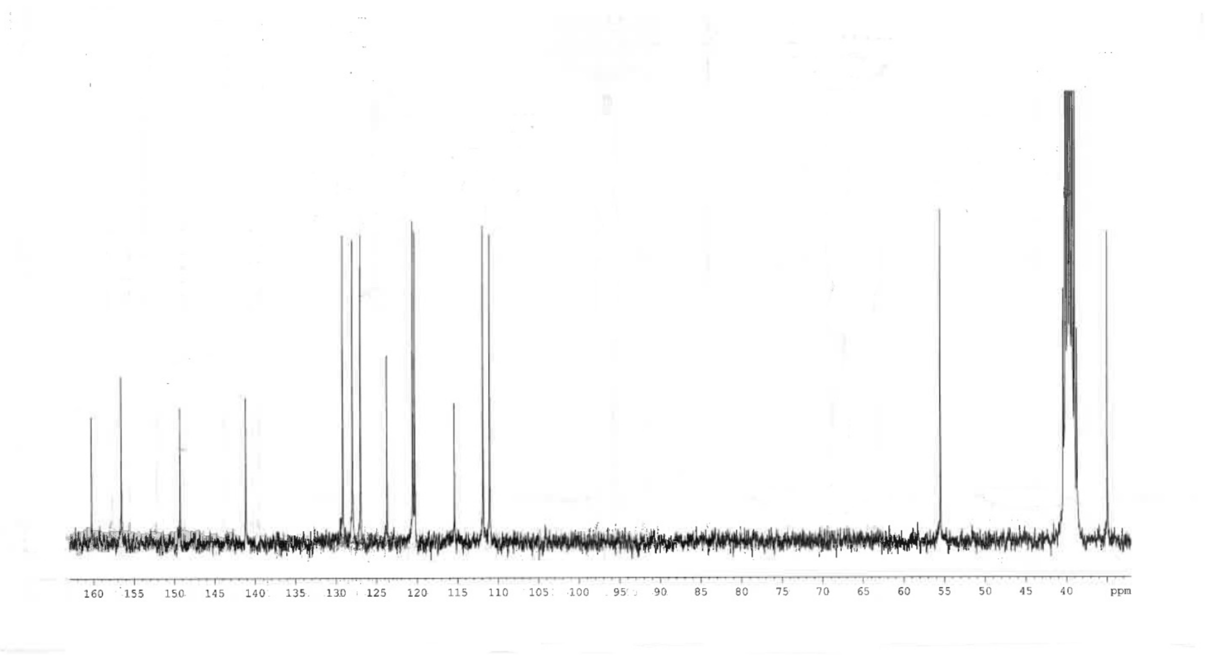

Figure S9. ^{1}H and ^{13}C NMR spectra of compound **15**.

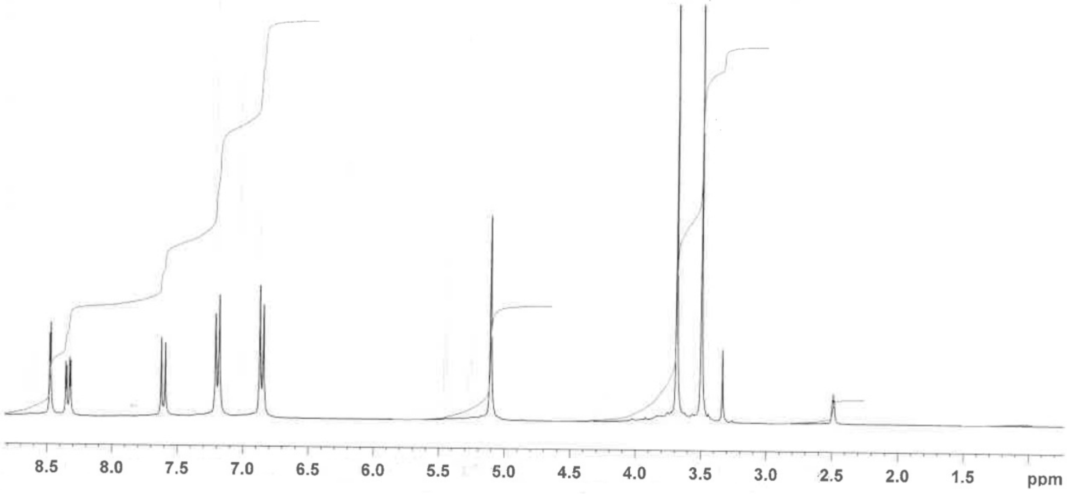
8.5 8.0 7.5 7.0 6.5 6.0 5.5 5.0 4.5 4.0 3.5 3.0 2.5 2.0 1.5 ppm

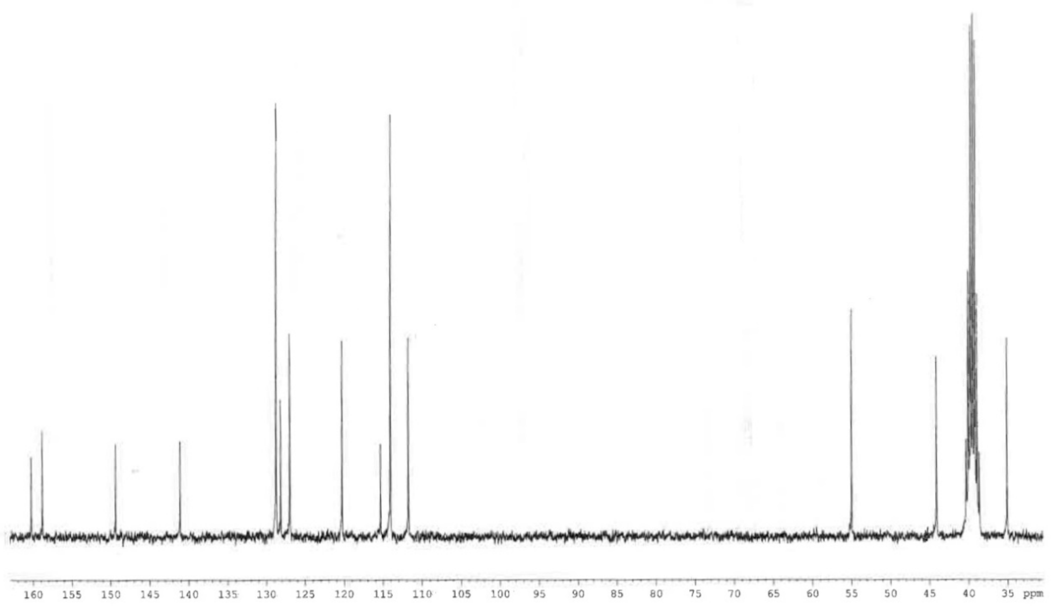

Figure S10. ^{1}H and ^{13}C NMR spectra of compound **16**.

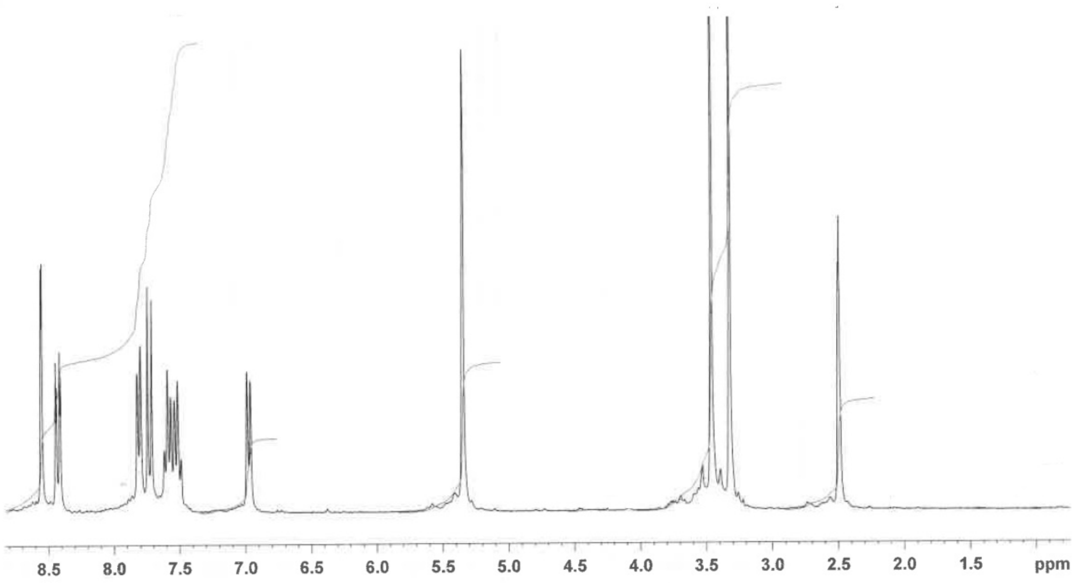
8.5 8.0 7.5 7.0 6.5 6.0 5.5 5.0 4.5 4.0 3.5 3.0 2.5 2.0 1.5 ppm

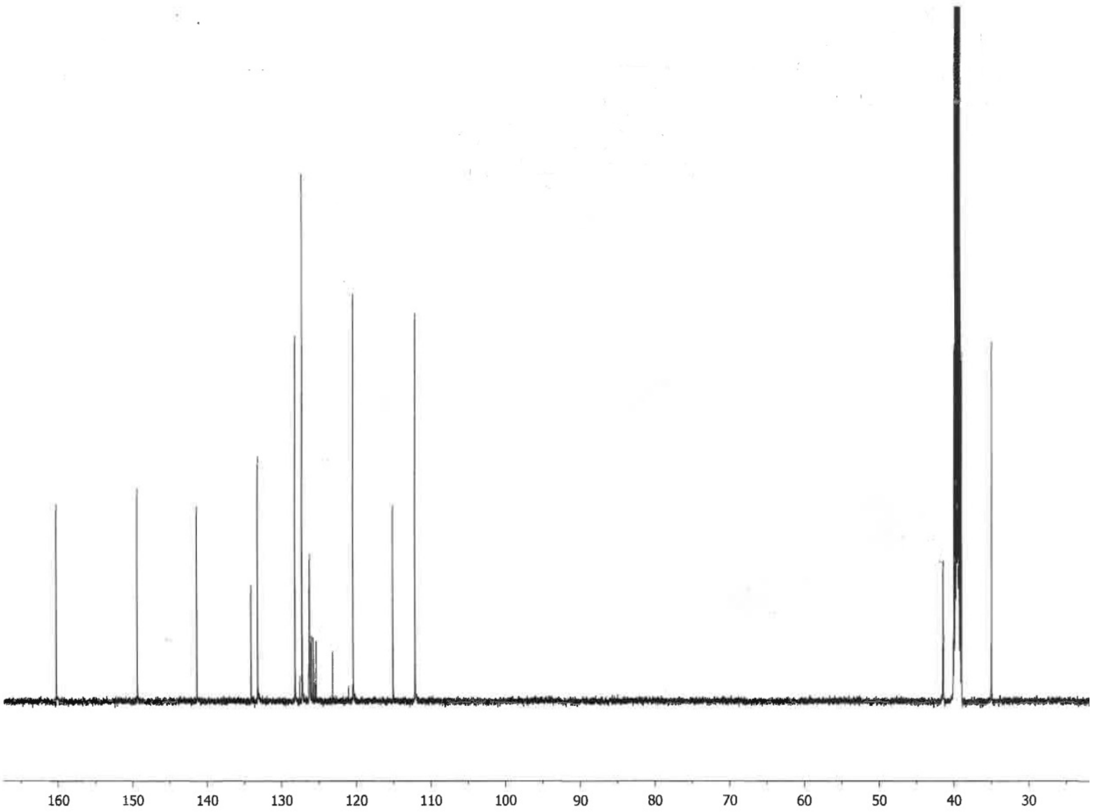

Figure S11. ^{1}H and ^{13}C NMR spectra of compound **17**.

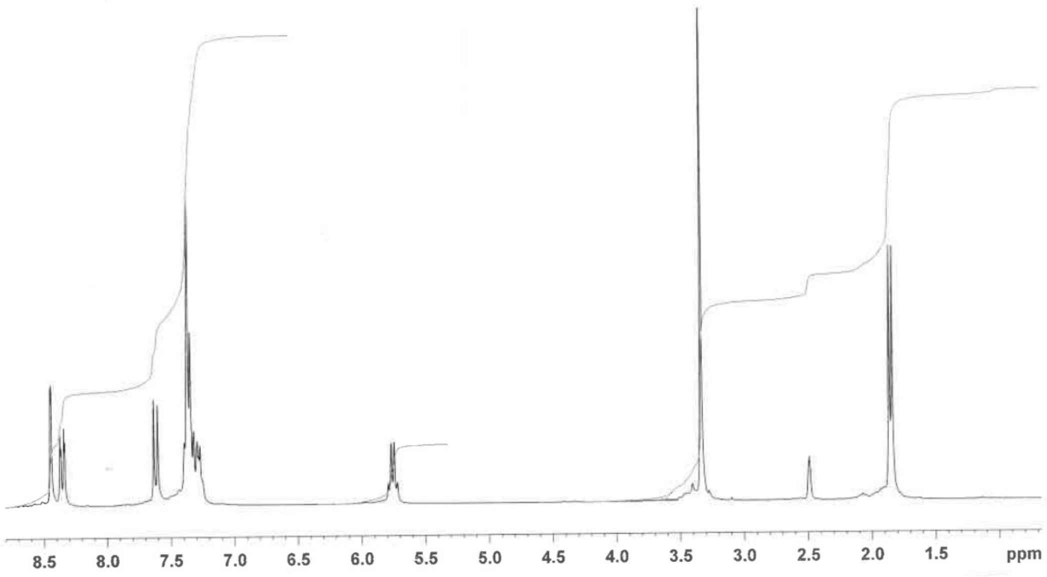
8.5 8.0 7.5 7.0 6.5 6.0 5.5 5.0 4.5 4.0 3.5 3.0 2.5 2.0 1.5 ppm

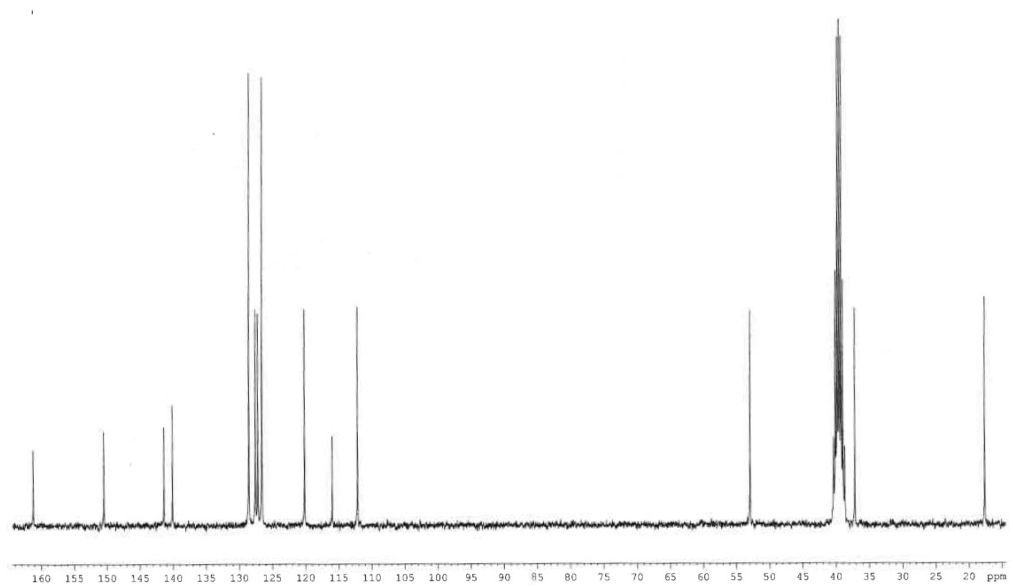

Figure S12. ^{1}H and ^{13}C NMR spectra of compound **18.**

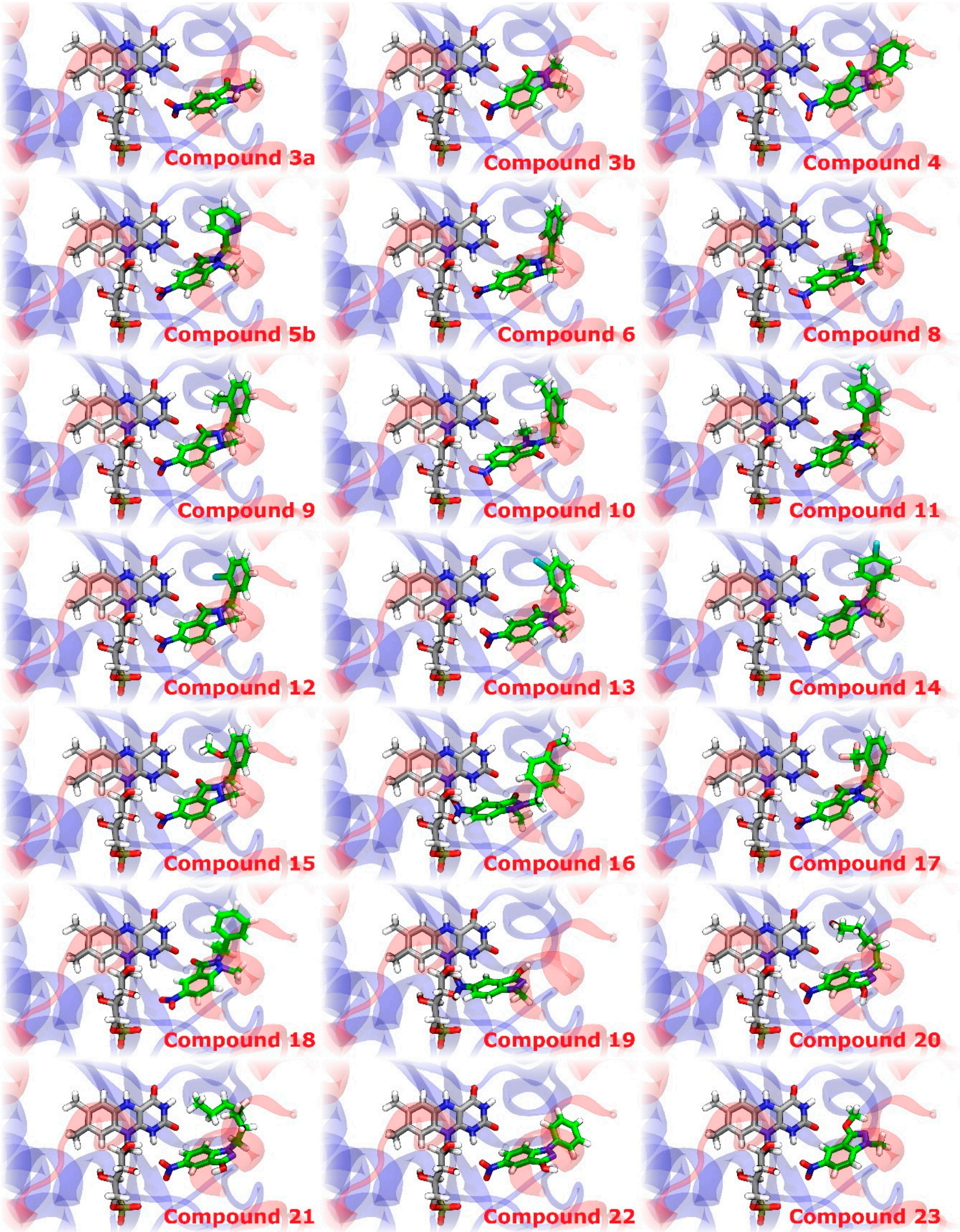

Figure S13. Binding modes of compounds **3a**, **b**, **4**, **5b**, **6** and **8** – **23** into the *tc*NTR binding site predicted through docking calculations.

Figure S14. IC_{50} values of RAW 264.7

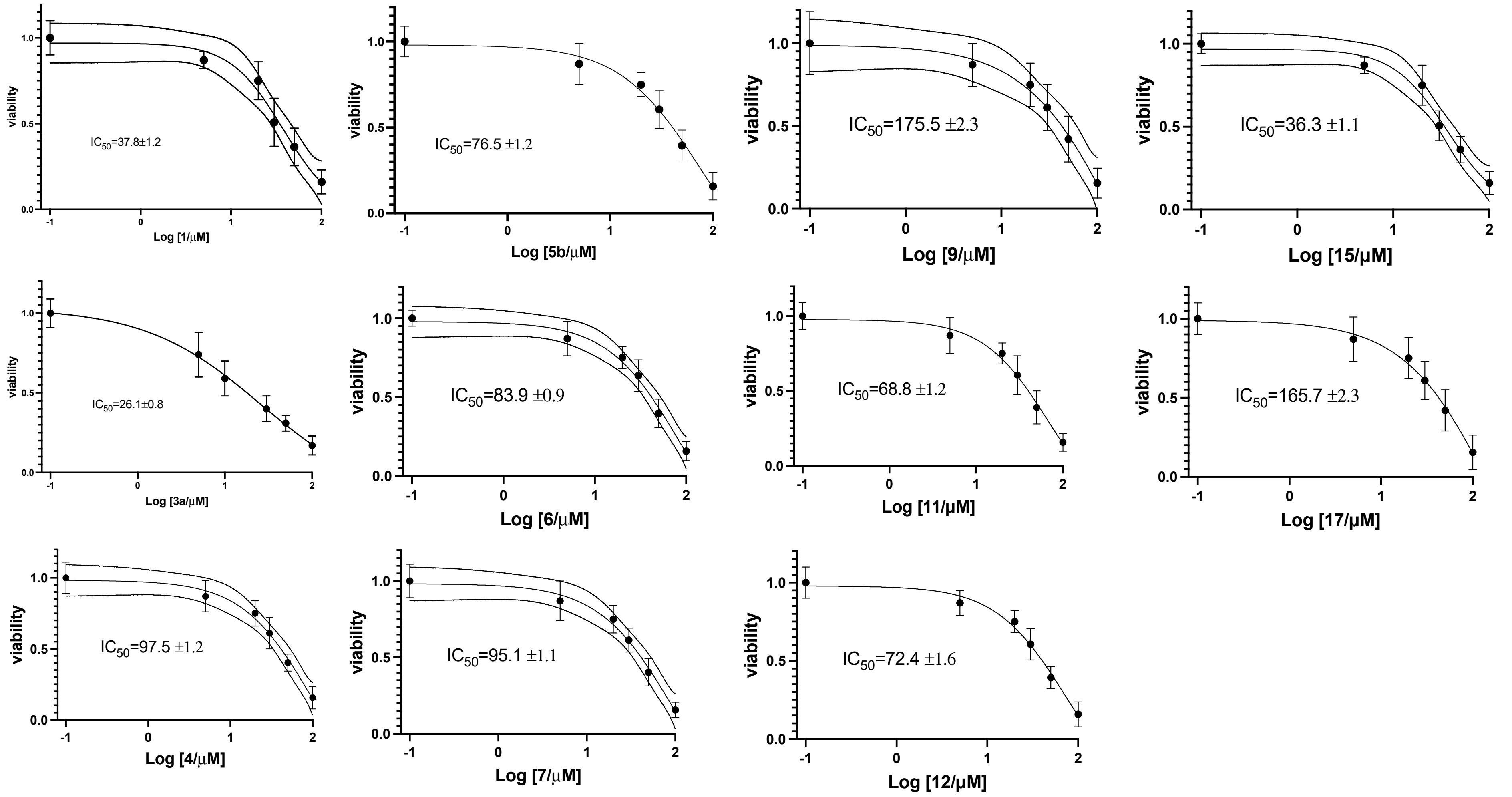

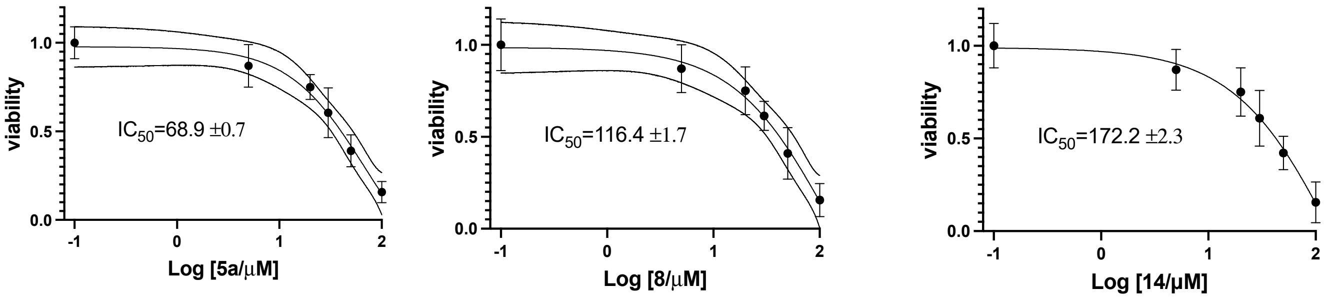

Figure S15. IC$_{50}$ values of Epimastigotes

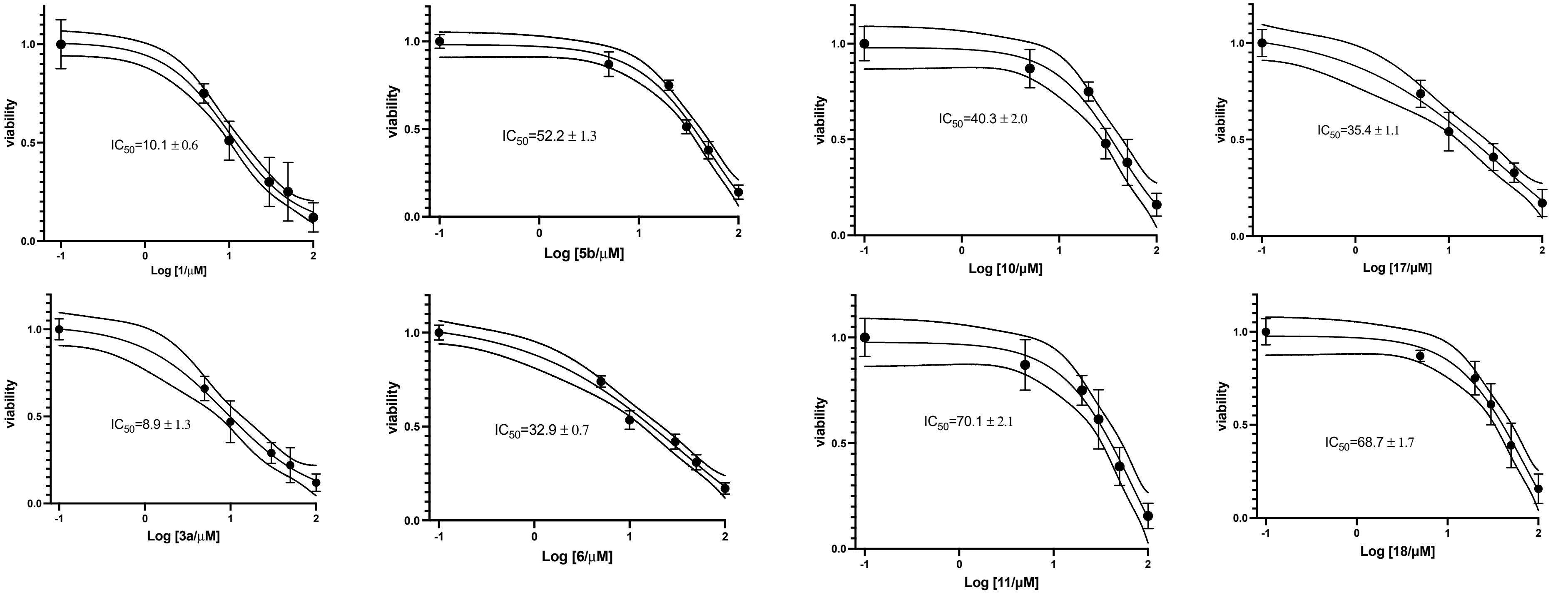

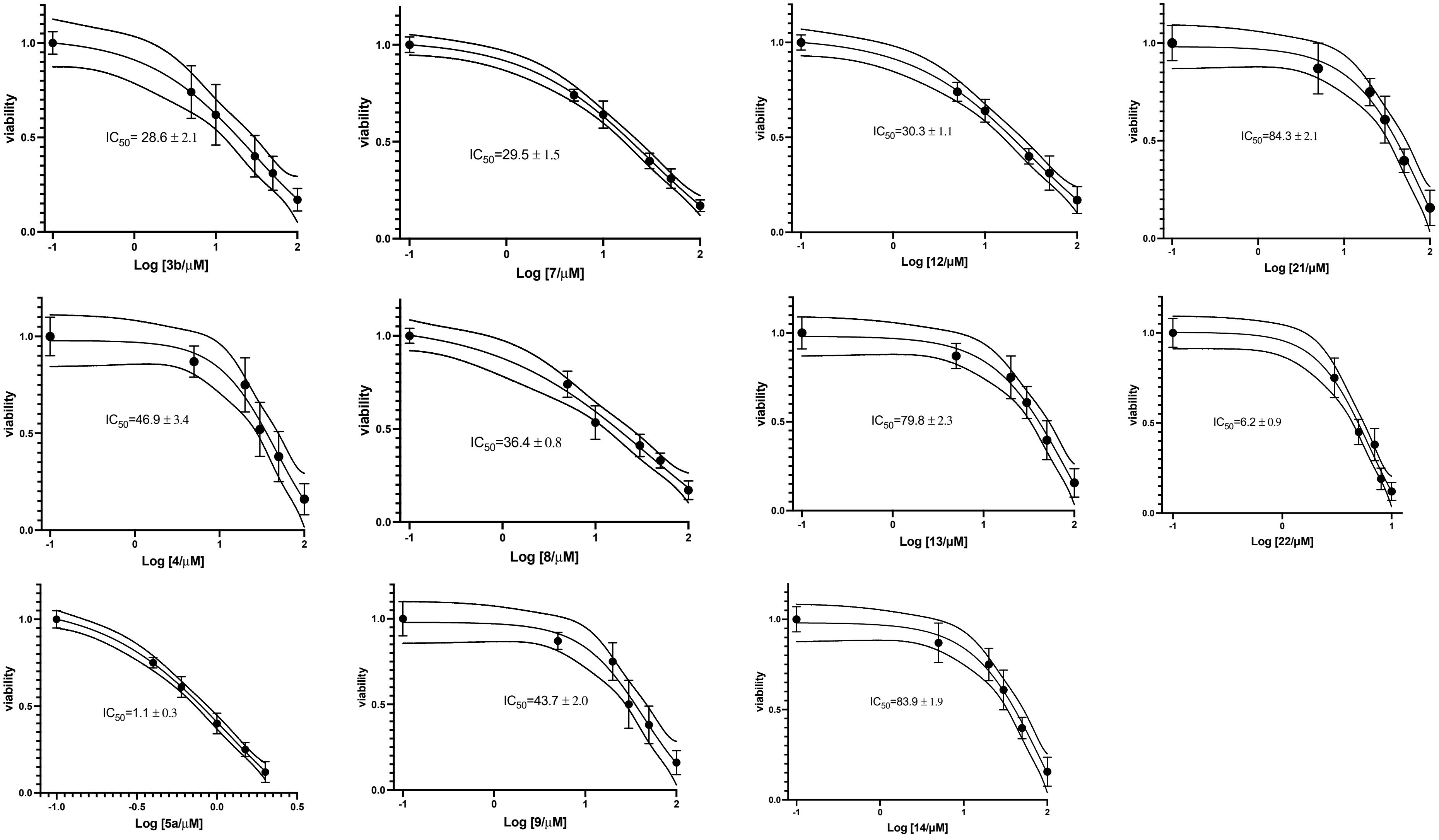

IC50= 28.6 ± 2.1
Log [3b/μM]
IC50=29.5 ± 1.5
Log [7/μM]
IC50=30.3 ± 1.1
Log [12/μM]
IC50=84.3 ± 2.1
Log [21/μM]
IC50=46.9 ± 3.4
Log [4/μM]
IC50=36.4 ± 0.8
Log [8/μM]
IC50=79.8 ± 2.3
Log [13/μM]
IC50=6.2 ± 0.9
Log [22/μM]
IC50=1.1 ± 0.3
Log [5a/μM]
IC50=43.7 ± 2.0
Log [9/μM]
IC50=83.9 ± 1.9
Log [14/μM]
viability

Figure S16. IC_{50} values of Trypomastigote

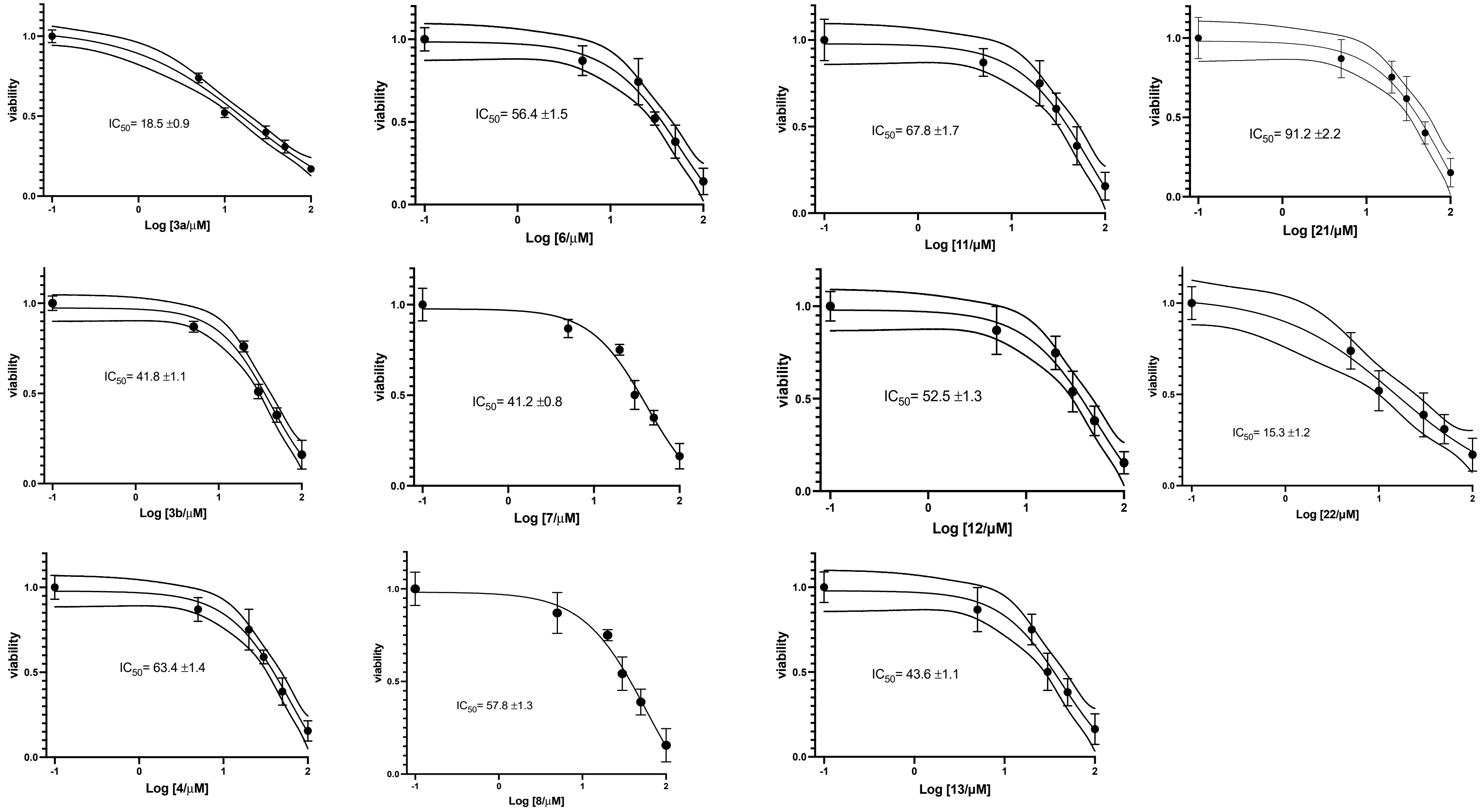

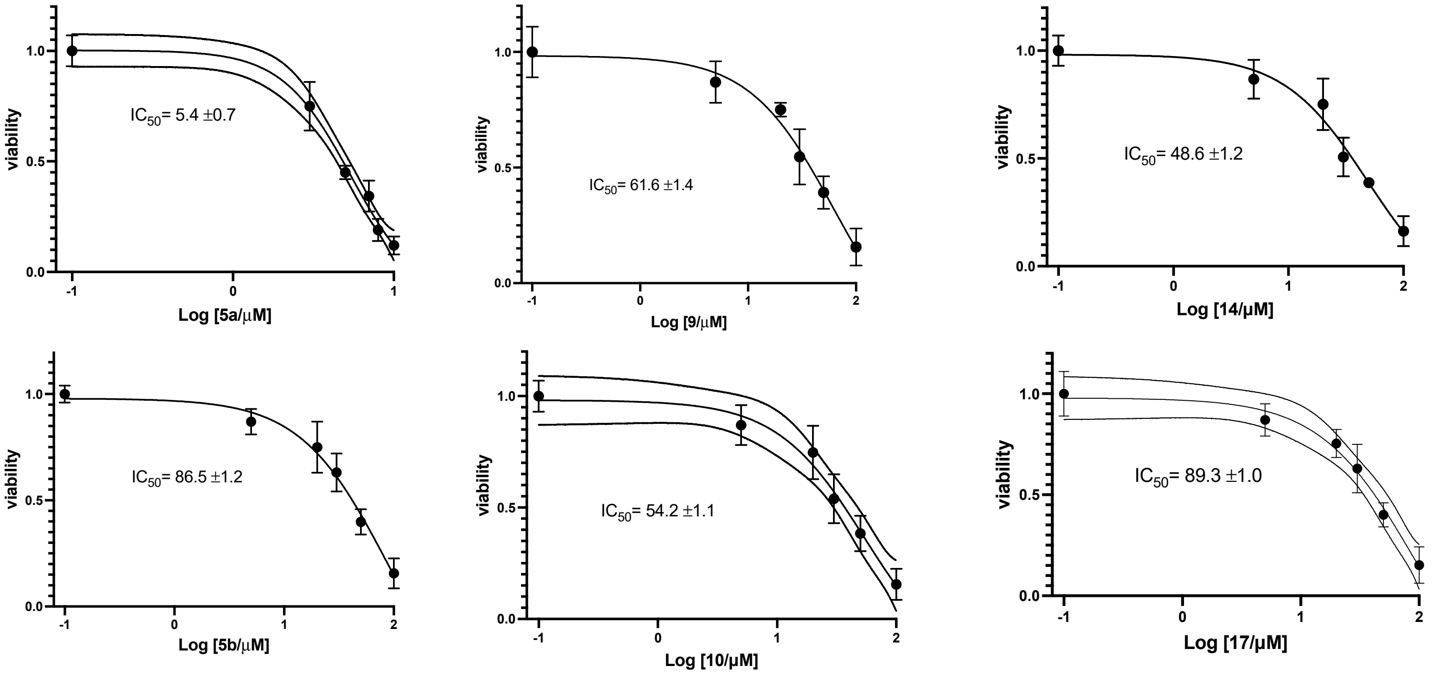
viability
IC50= 5.4 ±0.7
Log [5a/μM]
IC50= 61.6 ±1.4
Log [9/μM]
IC50= 48.6 ±1.2
Log [14/μM]
IC50= 86.5 ±1.2
Log [5b/μM]
IC50= 54.2 ±1.1
Log [10/μM]
IC50= 89.3 ±1.0
Log [17/μM]
